# Scanning Electron Microscopy Imaging of Twist Domains in Transition Metal Dichalcogenide Heterostructures

**DOI:** 10.1021/acsnano.4c09364

**Published:** 2024-12-06

**Authors:** Evan Tillotson, James G. McHugh, James Howarth, Teruo Hashimoto, Nicholas J. Clark, Astrid Weston, Vladimir Enaldiev, Sam Sullivan-Allsop, William Thornley, Wendong Wang, Matthew Lindley, Andrew J. Pollard, Vladimir I. Fal’ko, Roman V. Gorbachev, Sarah J. Haigh

**Affiliations:** † Department of Materials, University of Manchester, Manchester M13 9PL, U.K.; ‡ Department of Physics and Astronomy, University of Manchester, Manchester M13 9PL, U.K.; § National Graphene Institute, University of Manchester, Manchester M13 9PL, U.K.; ∥ National Physical Laboratory, Hampton Rd, Teddington TW11 0LW, U.K.

**Keywords:** twistronics, TMDs, ferroelectric domains, 2D material semiconductors, nanomaterials, electron microscopy

## Abstract

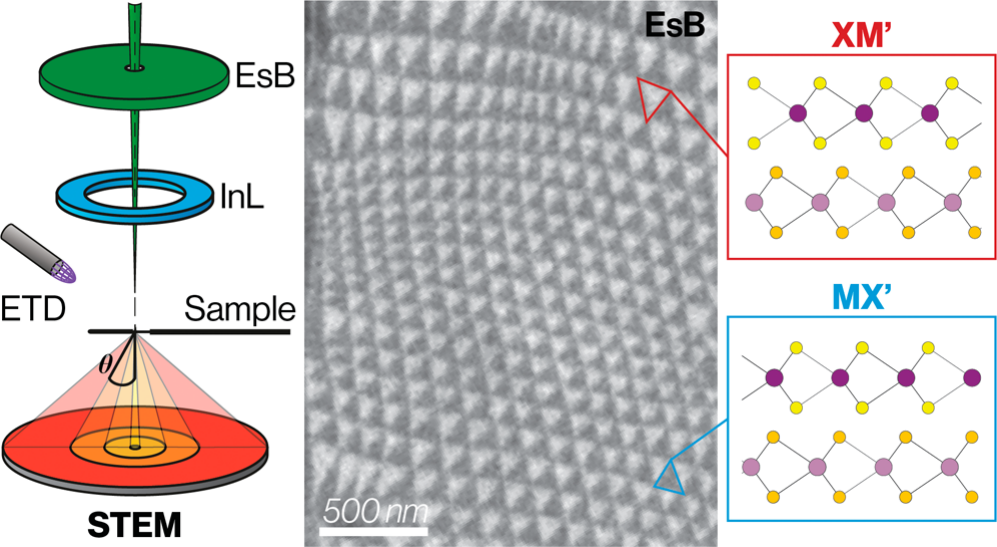

Twisted two-dimensional (2D) material heterostructures provide an exciting platform for investigating fundamental physical phenomena. Many of the most interesting behaviors emerge at small twist angles, where the materials reconstruct to form areas of perfectly stacked crystals separated by partial dislocations. However, understanding the properties of these systems is often impossible without correlative imaging of their local reconstructed domain configuration, which exhibits random variations due to disorder and contamination. In particular, visualization of the local domain configuration allows determination of the local twist angle and, hence, the local lattice strain. Here, we demonstrate a simple and widely accessible route to visualize domains in the as-produced twisted transition metal dichalcogenide (TMD) heterostructures using electron channeling contrast imaging (ECCI) in scanning electron microscopy (SEM). This nondestructive approach is compatible with conventional substrates and allows domains to be visualized even when sealed beneath an encapsulation layer. Complementary theoretical calculations reveal how a combination of elastic and inelastic scattering leads to contrast inversions at the specified detector scattering angles and sample tilts. We demonstrate that optimal domain contrast is therefore achieved by maximizing signal collection while avoiding contrast inversion conditions.

## Introduction

Van der Waals (vdW) heterostructures offer opportunities for materials design through controlling the relative twist angle between adjacent atomic layers. When 2D crystals in contact have a similar lattice constant and/or a small angular misalignment, a moiré superlattice forms at their interface.^1^ When the superlattice repeat period is long enough, the system can spontaneously reconstruct, forming pristine crystalline domains separated by narrow dislocations. For twisted superlattices, the structure and shape of the moiré pattern depends on the lattice mismatch and the relative twist angle between the two layers.^2^ The long-range periodicity of twist domains can dramatically affect the (opto)electronic properties of the system,^3^ and has become a topic of active research referred to as “twistronics”.^4−6^ The ability to tune the electronic structure through reconstructed twist domains has enabled studies of correlation effects in flat electronic bands,^7,8^ revealing exciting physical phenomena such as superconductivity in graphene,^9^ exciton trapping,^10^ resonant excitonic states^11,12^ and excitonic density waves.^13^ Unfortunately, both top-down mechanical assembly and bottom-up chemical synthesis of vdW heterostructures often result in large variability in the local twist angle and therefore large variability in the size of the reconstructed twist domains, even within single crystal samples.^14^ In the case of vdW assembly from exfoliated crystals, this inhomogeneity originates from random strain fields acquired during the nanofabrication process and manifests as approximately 0.1° twist angle disorder.^15^ This is problematic because the properties of reconstructed twisted 2D structures are sensitive to small local twist angle variation,^16^ making the correlation of lattice distortion and electronic properties necessary for the future progress of twistronics.^17^

To date, various techniques have been utilized for mapping the reconstructed domain networks in twisted heterostructures. Scanning tunneling microscopy (STM) allows high spatial resolution imaging via probing of the moiré-induced variations in the local electronic structure.^18,19^ Moreover, the technique can be used to map strain tensors and twist angles from experimentally acquired images.^20^ Other, less demanding, scanning probe techniques such as piezoresponse force microscopy allow sub-5 nm visualization.^21^ Aberration-corrected low energy electron microscopy (LEEM) can visualize the average atomic structure of domains and strain information.^22^ However, these techniques require the twisted interface to be at the surface. As the majority of 2D material devices require encapsulation,^23^ typically in hexagonal boron nitride (hBN), either due to their air sensitivity, to increase their (opto)electronic performance, or for electrostatic gating, an alternative nondestructive imaging approach is required.

Transmission electron microscopy (TEM) allows imaging of the microstructure of superlattices down to the atomic scale,^24,25^ as well as the calculation of local lattice strain from electron diffraction mapping of the moiré unit cell.^26,27^ Yet the technique requires suspended samples, which are often incompatible with property measurements, making it impossible to discern the dependence of the (opto)electronic properties on the local atomic configuration. Scanning near-field optical microscopy (SNOM) allows imaging of domain networks where they provide sufficiently strong confined light-matter interactions (polaritons), for example in twisted graphene through detecting plasmon reflections at domain boundaries^28^ or exploiting phonons in hBN.^29^ Nonetheless, this is a relatively specialized technique which requires artificial doping of the heterostructure, so is not generally applicable to unmodified optoelectronic device structures.^28−31^

Scanning electron microscopy (SEM) imaging has many advantages for imaging twist domains in 2D heterostructures, being a widely available, nondestructive, and surface sensitive technique compatible with most electronic device configurations. More specifically, electron channeling contrast, where an enhanced electron yield occurs at specific lattice orientations, has been widely applied for SEM imaging of crystal grain structures and dislocations in bulk materials.^32,33^ While SEM electron channeling contrast imaging (ECCI) has rarely been employed to visualize twisted 2D materials,^2,34,35^ a general understanding of the electron scattering processes arising from reconstructed 2D lattices is currently absent.

In this work we conduct a comprehensive SEM ECCI analysis of twisted TMD heterostructures. We analyze elastic and inelastic scattering contributions in both secondary electron (SE) and backscattered electron (BSE) detector signals and establish a complementary theoretical model to support our observations. We demonstrate the robustness of BSE ECCI imaging of reconstructed domains, being insensitive to stage rotation angle and which does not require sample tilting. Finally, we study the effects of sample thickness, choice of substrate, and hBN encapsulation. This work demonstrates the SEM ECCI method to be a widely accessible and practical imaging solution for the twistronics community.

## Results

**Figure 1:**
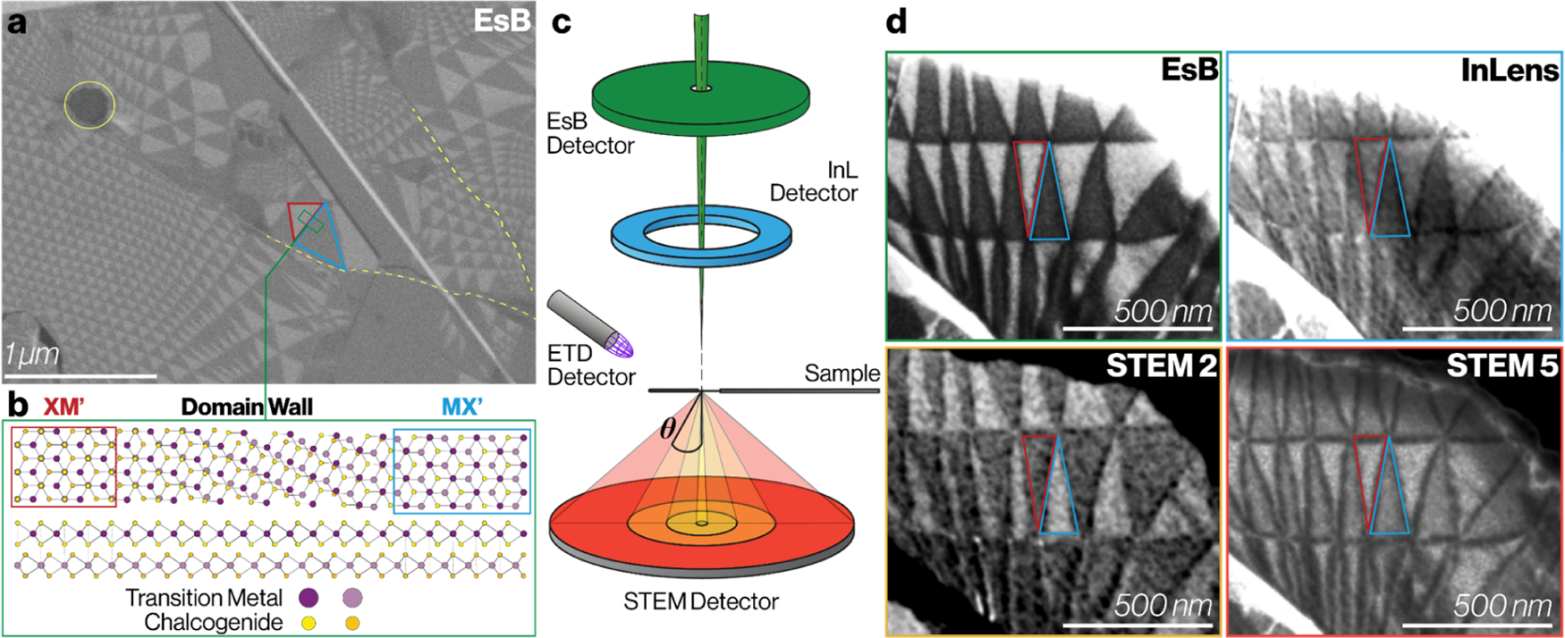
SEM image of reconstructed domains in parallel stacked twisted TMD bilayer. (a) SEM EsB detector image showing a typical twisted bilayer of MoS_2_ on a graphite/SiO_2_ substrate with visible disorder in the reconstructed domain network. Contamination pockets and wrinkles are shown via yellow circles and dashed lines, respectively. (b) Schematic illustrating the atomic structure within reconstructed 3R type domains (XM′ and MX′ stacked) and at the domain wall for parallel, marginally twisted TMD crystals. Top: plan view along [0001]. Bottom: cross-section viewed along [2–1–10]. In (a) examples of XM' and MX′ stacked domains are highlighted by the red and blue triangles, respectively. (c) Schematic showing the location of different detectors in the SEM relative to the sample, and the scattering angle θ. The STEM detector has 4 segments (STEM1, STEM2, STEM3/4, and STEM5) each having a different range of inner, outer collection angles, which can be slightly modified by changing the distance between the detector and the sample. (d) Comparing simultaneously acquired images of twisted superlattice domains in a suspended MoS_2_ bilayer acquired with the EsB, InL, STEM2 (low angle transmitted electron) and STEM5 (high angle transmitted electron) detectors. Positive domain contrast is defined as where *I*_XM –_*I*_MX_ > 0 (red XM'domains brighter as in EsB, InLens and STEM5 images) and negative domain contrast is where *I*_XM_ – *I*_MX_ < 0, (blue MX' domains brighter, as in STEM2 image).

When two TMD monolayers are aligned close to parallel orientation of the unit cell (i.e., with a small angular mismatch) the lattice reconstruction results in the emergence of tessellated triangular domains (see Figure 1a).^36^ The two alternating domain types are both rhombohedral (3R stacking) but are (0001) mirror symmetry equivalent, with one denoted MX′ (metal atoms in the top layer located above the chalcogen atoms in the lower layer) and the other XM′ (vice versa). These stacking domains are separated by several-nanometer-wide domain walls which constitute a partial dislocation, illustrated schematically in Figure 1b. In all samples studied, mechanical transfer methods used during fabrication resulted in a noticeable variation in both domain periodicity and size due to the presence of random local strain and pockets of interlayer contamination.^37^ Examples include wrinkles (white or dark lines) and contamination pockets (dark areas) which locally distort the triangular domain network. Both of these effects can be seen in the SEM channeling contrast image in Figure 1a, acquired with an energy selective backscatter (EsB) electron detector. Here, an MoS_2_ twisted bilayer was assembled using the tear-and-stack approach^38^ and subsequently placed on a relatively thick (∼50 nm) graphite crystal which had been exfoliated onto an oxidized silicon wafer.

### Domain Contrast in Suspended Samples

To deconvolute the complex picture of electron scattering and detection, we first focus our attention on freely suspended TMD bilayers where the substrate-induced scattering is absent. To fabricate these samples, twisted bilayers have been deposited onto few-nm thick hBN membranes with predrilled apertures and then placed over the holes in silicon nitride TEM grids (see methods and Supporting Information Section 1 for full fabrication details). For our analysis, an archetypal SEM system (Zeiss Merlin Gemini II SEM) is used. Like most modern SEMs, this instrument is fitted with a variety of electron detectors both above and below the sample (Figure 1c). The most common detector in SEM imaging is the conventional Everhart-Thornley detector (ETD). This has a positive bias and is positioned at a relatively large distance from the scanned electron beam. ETD images are often termed “secondary electron” (SE) images but could more accurately be referred to as low energy electron images, since they can include information from any low energy electrons emitted from the sample surface. The maximum energy of these collected electrons varies with detector bias but is typically <100 eV.^39^ The other common SEM imaging mode is referred to as backscattered electron (BSE) imaging, and is achieved using an annular detector placed directly above the sample. This type of detector uses a negative applied bias, which serves to repel electrons with lower energy, ensuring only high energy electrons emitted from the sample contribute to the signal. The Zeiss Merlin Gemini II SEM has two such detectors: the annular in-lens (InL) and Energy selective Backscattered (EsB) detectors, being distinguished by their different angular ranges relative to the incident electron probe. The SEM in this study also has a segmented scanning transmission electron microscopy (STEM) detector below the sample, which allows electron transparent regions to be studied in transmission mode. The detector collects electrons of any energy, and is split into 4 concentric annular segments with STEM1 being the central circle, and STEM2, STEM3/4 and STEM5 ring detectors with increasing radii as illustrated in Figure 1c. Note that the STEM3/4 detector is split into orthogonal pairs of quadrants and pairs can be read separately as STEM3 or STEM4 to allow differential phase contrast (DPC) STEM imaging. However, for the purposes of this work we employ both sides of the detector together to create a single annular detector.

Figure 1d shows simultaneously acquired SEM images for a freely suspended sample region consisting of a MoS_2_ twisted bilayer with incident beam direction close to [0001], where reconstructed moiré superlattice domains are clearly visible as triangular regions with alternating contrast. We use an accelerating voltage of 1500 V and a compromise working distance of ∼6 mm (we discuss the reasons for these settings later in the manuscript). To quantitatively compare the domain contrast for different detectors we use the Michelson contrast, calculated from measurements of the average intensity in the image of XM′ and MX′ domains, *I*_XM_ and *I*_MX_ respectively, and defined as (*I*_XM_ – *I*_MX_)/(*I*_XM_ + *I*_MX_). Our previous work allows us to determine that for these SEM imaging conditions and in the absence of sample tilt, XM′ stacked domains are brighter than MX′ domains in EsB images, based on both their response to a perpendicular applied electric field and their increased tunneling current measured using conductive atomic force microscopy.^36^ Here we will define this as positive contrast, *I*_XM –_*I*_MX_ > 0, while the STEM2 detector image shows negative contrast, *I*_XM_ – *I*_MX_ < 0. For further details of the method used for quantifying the domain contrast from the SEM images see SI Section 2 and Figures S2.1–3.

In Figure 1d, the greatest domain contrast is observed for the EsB detector (Michelson contrast of 21%), followed by the InL detector (20% contrast). The ETD image is not shown as insufficient signal was detected from the freely suspended sample to generate an image above the noise. Out of all the STEM detector segments, STEM2 and STEM5 showed the greatest domain contrast (−12.4 and 11.7%, respectively). We now seek to understand the origin of the surprising contrast inversion, whereby in the STEM2 the bright and dark domains are reversed compared to the EsB, InL and STEM5 images. Flipping the specimen upside down causes the domain contrast of all images to invert as expected (since MX′ domains then become XM′ when inverted and vice versa, see Figure S2.5).

**Figure 2:**
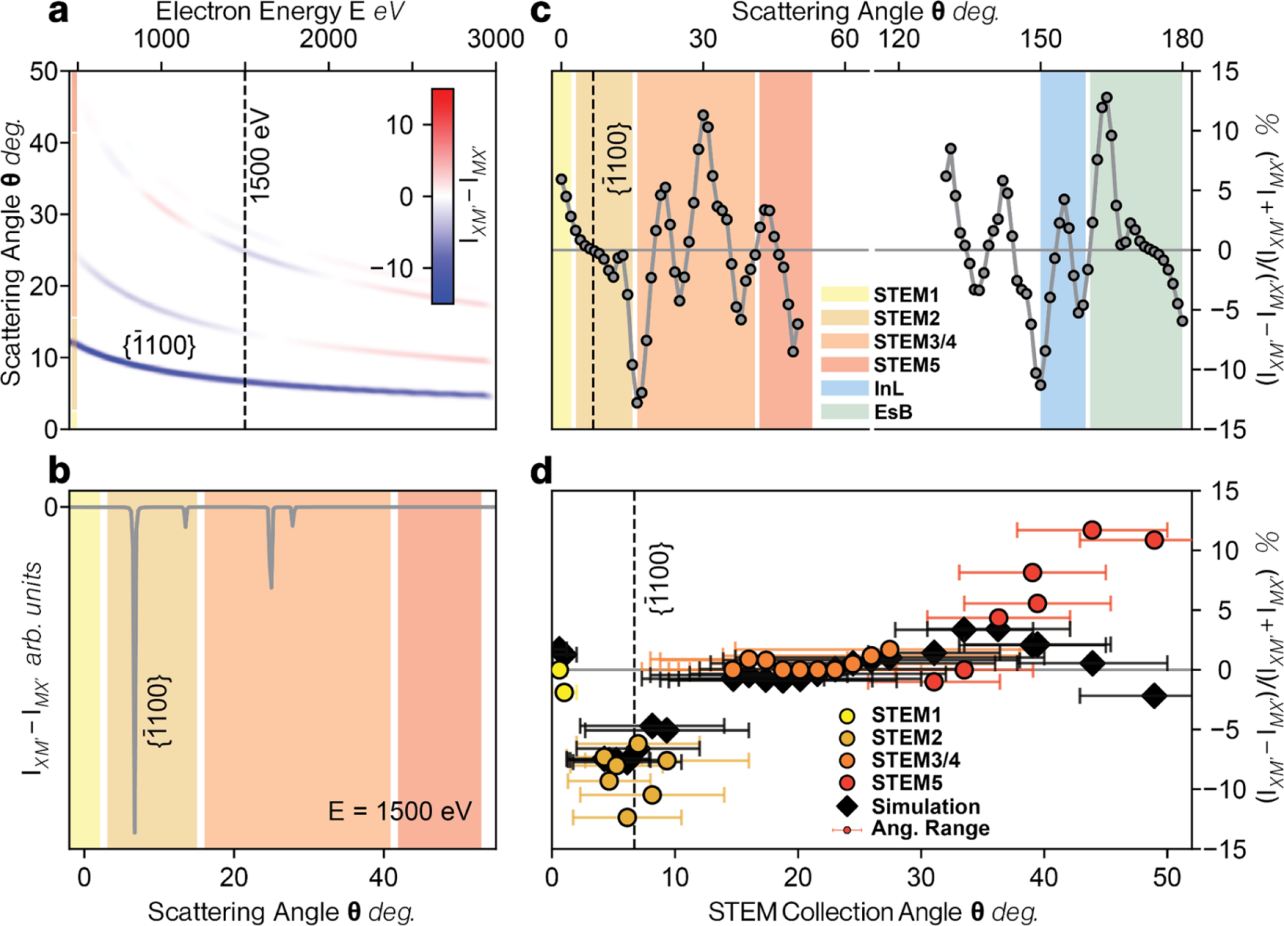
Theoretical estimation of domain contrast in freely suspended samples and comparison to experimental data. (a) The difference in the elastic Bragg scattering transmitted intensity for the two domains, *I*_XM_ – *I*_MX_ as a function of primary electron beam energy *E* (eV) and the scattering angle, θ. A color scale is used to show the scattering intensity where white equates to no intensity. The dashed vertical line highlights *E* = 1500 eV, the value used to the collect the experimental data points in (d). (b) *I*_XM_ – *I*_MX_ for *E* = 1500 eV as a function of the scattering angle θ for elastic scattering. (c) Domain contrast (*I*_XM_ – *I*_MX_/*I*_XM_ + *I*_MX_) as a function of scattering angle for inelastically scattered electrons (note that angles greater than 90° represent reflected inelastically scattered electrons). Signal is radially averaged over both crystallographic directions for generated electrons with *E* = 100 eV and a primary beam energy *E*_0_ of 1500 eV. Color shading in (b) and (c) matches the approximate angular ranges of the SEM detectors. (d) Domain contrast from STEM detectors for a freely suspended twisted MoS_2_ bilayer sample. Each data point shows the mean domain contrast in a particular image with the angular range of scattering angles accepted by the detector, Δθ, shown by the horizontal bars. The camera length has been adjusted to change the annular ranges of the 4 STEM detectors while imaging the same specimen area. Vertical error bars representing the variation of the measured contrast for different domains within the images are not shown as these were all smaller than the data markers. The points are horizontally positioned and color coded to match the STEM detectors used to acquire the image whose relative positions are shown in Figure 1c. The black data points are those calculated from theoretical modeling of the combination of elastic and inelastic scattering (using the same angular ranges as the equivalent experimental data point). The angle of the {1̅100} Bragg reflection at an incident electron energy of 1500 eV is indicated by the dashed vertical line.

First, we consider the domain contrast inversion seen in Figure 1d when comparing the transmitted (STEM) detector signal at low angle (STEM2) and high angle (STEM5), to better understand the underlying electron scattering processes. To measure the domain contrast as a function of scattering angle, θ, (see Figure 1c) we vary the annular range of the scattering angle, Δθ, collected by each STEM detector segment by adjusting the sample–detector distance. The results are presented in Figure 2d, where each horizontal bar shows the integral contrast value for electrons scattered within an interval between θ_1_ and θ_2_, as denoted by the horizontal position of the bar. The greatest contrast is seen for collection angles in the range 4–12° (negative contrast) and the range 24–50° (positive contrast).

To understand the unusual contrast behavior, and specifically the zero-contrast inversion point at ∼20°, we have performed theoretical calculations of both elastic and inelastic scattering contributions to domain contrast. We first consider purely elastic scattering, during which the transmitted electrons undergo Bragg reflections. The resulting Bragg peaks, g, for XM′ and MX′ stacked domains can be expected to have different intensities, referred to as *I*_XM_(g) and *I*_MX_(g) respectively. The relative magnitude of *I*_XM_(g) and *I*_MX_(g) for each Bragg reflection (*I*_XM_ – *I*_MX_) varies as a function of the incident electron beam energy, *E* (see Figure 2a). At the experimental beam energy of 1500 eV, the first g = {1̅100} Bragg peak occurs at θ ≈ 7° with our calculations predicting that this will give strong negative domain contrast (see Figure 2b), which clearly correlates to the negative domain contrast values observed for the STEM2 detector with an angular range of 1.2° < θ < 16°. However, higher order Bragg reflections are also predicted to give negative domain contrast, so elastic scattering alone cannot explain the observed contrast inversion for θ ≈ 20°. That said, sign reversal is predicted for elastic contributions to STEM images at lower or higher incident beam energies, for example Figure 2a shows that at an incident beam energy of 1000 eV the Bragg peak at ∼8° is negative but the peak at ∼30° is positive (see SI Section 3.1 for a full discussion and details of numerical calculations).

As contrast inversion cannot be explained by elastic scattering only, we must therefore consider the contribution from the electrons that are inelastically scattered. A semiclassical model of channeling contrast was employed, where generation and attenuation of secondary electrons are both proportional to electron density. Secondary electrons are considered to be generated by the primary beam, proportional to the local electron density of an MoS_2_ bilayer, *p*_gen_ ∝ ρ(*r*) and the corresponding electron–electron cross-section for a primary beam of energy *E*_0_. Inelastic scattering of the primary beam transfers energy, Δ*E*, to lattice electrons in the sample. The probability for an electron to escape because of this process is then taken as the integral along the escape path, with a cross-section corresponding to the transferred energy, Δ*E*. Cross sections were taken to reproduce the empirical TPP-2 M mean-free paths,^40,41^ which largely accounts for low-energy valence band scattering (plasmon and interband transitions) and does not fully incorporate large energy transfer and high angle deflections (see SI Section 3.2 for full model details and SI Section 3.2.1 for discussion of inelastic cross sections).

Using this model, the inelastic scattering behavior variation is computed as a function of scattering angle, θ, along a given in-plane crystallographic direction (zigzag, ϕ = 60°, or armchair ϕ = 30°) as shown in SI Figures S3.11 and S3.12, respectively, for an incident energy of 1500 eV and for generated electrons with Δ*E* = 100 eV. The radially averaged sum is plotted in Figure 2c and shows a trough corresponding to maximum negative domain contrast at ∼16°, with peaks corresponding to maximum positive domain contrast at ∼22 and ∼30°. It is therefore possible to reproduce a good match to the experimentally observed STEM detector contrast behavior for scattering angles up to θ ∼ 35° by combining elastic and inelastic scattering contributions with an empirically determined weighting based on a best fit to the experiment, illustrated by the black dots in Figure 2d. The annular dependence of secondary electrons with higher energy losses also shows oscillatory behavior and is in phase with the Δ*E* = 100 eV data as shown in Figure S3.15. The discrepancy between the experiment and theory at higher scattering angles can be understood as a result of plural scattering of electrons or due to large energy loss associated with ionization of core electrons (binding energies, 200–500 eV), neither of which were considered in this model (as discussed in SI Section 3.2.4).

Similar modeling can also explain the features observed in the reflected signals for suspended samples. The elastic signal reflected in the suspended bilayer (90° < θ < 180°) is 4 orders of magnitude smaller than the transmitted elastic signal so its contribution can be ignored (see SI Figure S3.7). Figure S3.13 shows the low energy secondary electron signal for suspended samples as a function of scattering angle, θ, which oscillates in a similar manner to the transmitted signal and so provides either positive or negative domain contrast depending on the angular range of the collected electrons. The EsB and InL both collect inelastically scattered electrons close to perfect reflection in the range 160° < θ < 179° and 150° < θ < 160° for the EsB and InL, respectively. In both angular ranges the domain contrast of the inelastic electrons is predicted to be positive; in agreement with experimental observations, although we note that for a full treatment, high energy inelastic scattering by core electrons should be included in the calculations. As the InL detector always gave similar images to the EsB detector, but with slightly lower contrast, from this point we focus on comparing ETD and EsB detector images.

The absence of visible contrast in the ETD image is assigned to the combination of specimen geometry, the low number of secondary electrons generated in such a thin suspended sample (1.4 nm) and the relatively higher mean energy of these secondary electrons compared to when the sample is thicker, which can be expected to result in weaker channeling behavior. While these calculations have been performed for MoS_2_ bilayers, we find similar contrast dependencies for WS_2_ bilayers, although the Michelson contrast values for WS_2_ are approximately 2–3 times higher than those of MoS_2_, which we assign to the higher atomic number promoting greater inelastic scattering. It is notable that walls between reconstructed domain walls appear dark in all reflected electron detectors and high scattering angle transmitted images (STEM5) independent of sample orientation (Figures 1d and S2.5). However, the domain wall contrast inverts in the STEM2 detector when the sample is flipped upside down, suggesting this contrast is due to Bragg diffraction effects. The magnitude of this domain wall contrast has been quantified as 3.7, 5.5, 8.4 and 4.6% for the EsB, InLens, STEM2 and STEM5, detectors respectively (the Michelson contrast has been measured relative to the local domain with the closest intensity and averaged across both rows of images in Figure S2.5). Domain wall contrast disappears in the presence of a support, suggesting that the contrast is the result of local crystal tilting due to the concentration of lattice strain at the boundary between domains.

### Domain Contrast in the Presence of a Substrate

**Figure 3:**
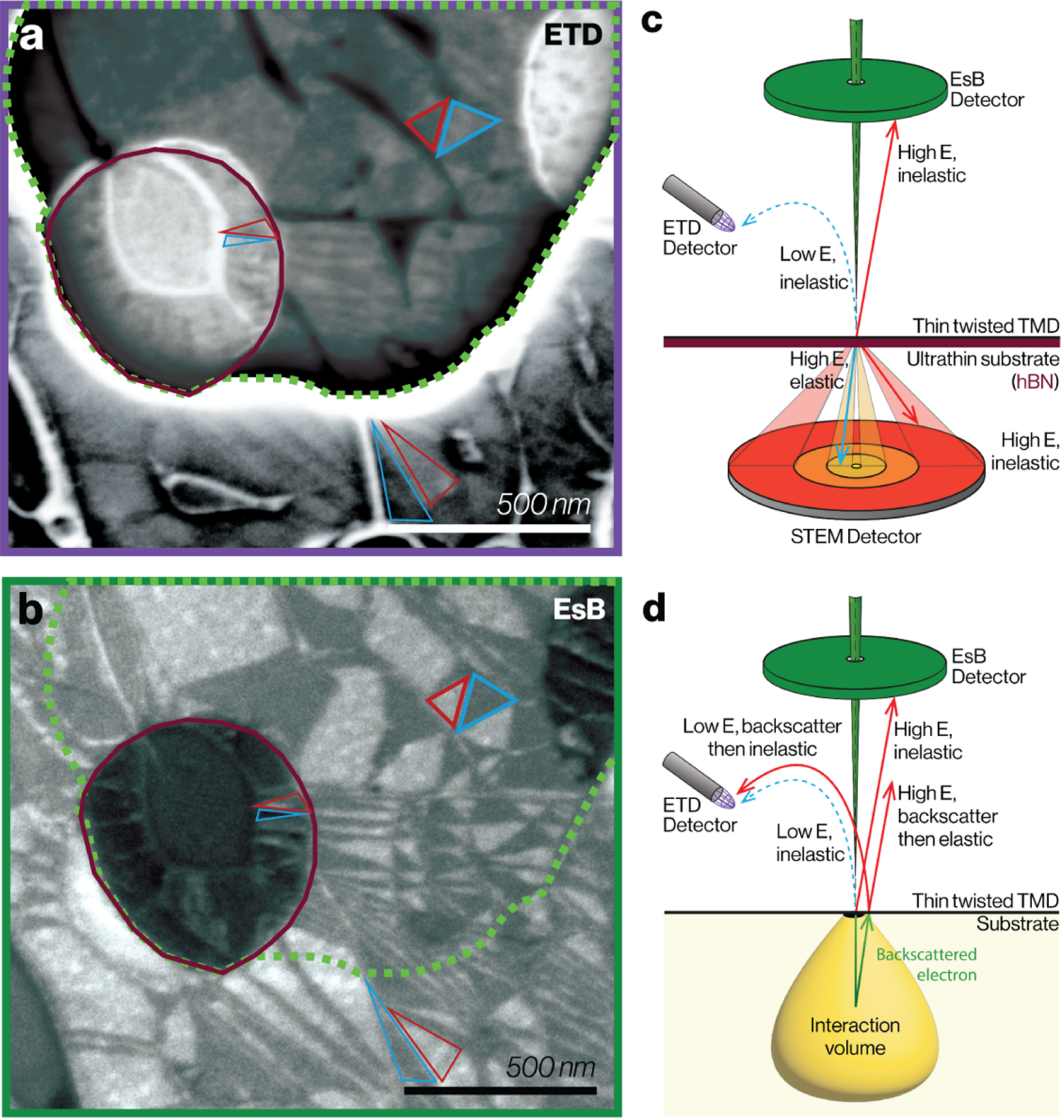
Comparing domain contrast between images acquired with ETD and EsB electron detectors. (a) and (b), ETD and EsB images, respectively, for a twisted WS_2_ bilayer sample which contains both substrate-supported and suspended regions. The maroon circular outline highlights a region where the bilayer is fully suspended and the area between the maroon line and the green dashed line indicates where the bilayer is on an ultrathin substrate (5 nm hBN). The area outside the green dashed line is where the sample is on both 5 nm hBN and a μm-thick SiN support. In (a) the ETD image is a montage of two images acquired with different dynamic range (optimized for the suspended region and fully supported regions, respectively, where the separate raw images are in SI Figure S2.8). (c) and (d), Schematic depiction of detector geometry and measured contrast for (c) suspended and (d) bulk substrate-supported twisted TMD samples. Arrows denote experimental domain contrast, which is labeled as positive (*I*_XM_ – *I*_MX_ > 0, blue) or negative (*I*_XM_ −*I*_MX_ < 0, red).

While suspended bilayers can provide high contrast for domain imaging, it is often essential to investigate heterostructure samples supported on a substrate, where only reflected signals are available in the SEM. Bulk substrates are needed to provide mechanical support, back gating and for integration of heterostructures into manufacturing processes. To understand the effect of the substrate on image contrast, Figure 3a,b compares simultaneously acquired ETD and EsB images where the field of view contains regions where the twisted bilayer is (i) fully suspended (inside the maroon circles), (ii) on an ultrathin support (outside the maroon circle but inside the dashed green line) and (iii) on a bulk support (lower part of the images outside the green dashed line). The presence of an ultrathin substrate (5 nm hBN) generates negative domain contrast in the ETD signal and enhances the positive domain contrast in the EsB signals compared to the fully suspended region. Replacing the ultrathin hBN with a bulk substrate (5 nm hBN on top of 1 um SiN) adds an additional background contribution to the EsB signal, slightly lowering the domain contrast relative to the ultrathin case. However, surprisingly, we find the domain contrast of the ETD image inverts to show positive domain contrast for the bulk substrate (further detail in SI Figure 2.4). Consequently, the ultrathin substrate gives inverted domain contrast for the ETD when compared to the EsB images, while the same contrast sign was always observed for all the EsB and ETD image pairs for twisted TMD layers on bulk supports. We note that similar bulk behavior was observed irrespective of whether the support was 1 μm SiN or 100 nm graphite on SiO_2_/Si wafers (see SI Figure S2.6).

The contrast generated by thin and thick substrate conditions is shown schematically in Figure 3c,d, respectively. To understand the domain contrast behavior of supported samples, it is necessary to consider both (i) the interaction of the incident electron beam with the sample (as discussed in the first part of this work) and (ii) the interaction of the electrons that inelastically backscatter in the substrate and transmit through the bilayer in the opposite direction. Electrons backscattered from the substrate emerge through the sample and are detected by the EsB and ETD detectors. We can therefore reuse the learnings from the STEM detector modeling but with two major differences. First, the backscattered electrons move through the bilayer in the opposite direction, so XM′ and MX′ domains now are inverted, producing opposite contrast sign in the elastic Bragg and inelastic contributions. Second, the backscattered electrons will have a wider spread of energies and angles than the primary beam. For elastic scattering, assuming an energy range of 500–1500 eV in the backscattered electrons, gives an angular distribution of the most significant {1̅100} Bragg reflection in the range of 168° < θ < 173°, which is within the angular range of the EsB detector. These backscattered electrons that subsequently Bragg scatter as they pass back through the bilayer will therefore have positive domain contrast, adding to the positive EsB contrast from the suspended sample.

For a flat sample, the ETD collects electrons leaving the detector at a wide range of angles,^42^ and therefore our calculations in Figure 2c show that both positive and negative contributions are possible depending on the angular range of the collected signal (which varies with the instrument settings and is difficult to quantify). We hypothesize that the angular range gives a negative contrast signal and that the ultrathin support increases the number of low energy electrons so that the negative domain contrast is visible above the detector noise in the ETD signal. For bulk supports the contrast inverts because this weak negative domain contrast signal is overwhelmed by the larger positive domain contrast due to the difference in the populations of the low energy electrons being generated as a secondary signal from the higher energy electron’s positive contrast (illustrated schematically in Figure 3d).

### Maximizing Contrast for Twist Domains

One of the main instrument parameters for optimizing SEM image contrast is the working distance (WD), which is the separation between the sample and the final probe forming lens.^43^ Investigation of the effect of WD on domain contrast shows the behavior that is expected for any SEM sample (SI Figure S4.1a,b). A WD of 7 mm provided the highest domain contrast for the ETD, while a slightly smaller value of 5 mm provided better contrast for the EsB image, with no contrast inversions observed for all the WD that were achievable. This suggests that the greatest domain contrast is achieved when the WD is set to maximize the number of collected electrons. A larger working distance improves the ETD detector collection efficiency but decreases the collection efficiency of the EsB and InL detectors. Smaller working distances favor higher spatial resolution as aberrations are reduced.^43^ The optimal accelerating voltage for achieving the highest contrast for 3R twist domains in MoS_2_ was found to be 1500–2000 V, with 1500 V used throughout this work for consistency. Further detail of optimizing SEM imaging conditions is given in SI Section 4.

### Domain Contrast as a Function of Sample Tilt

**Figure 4:**
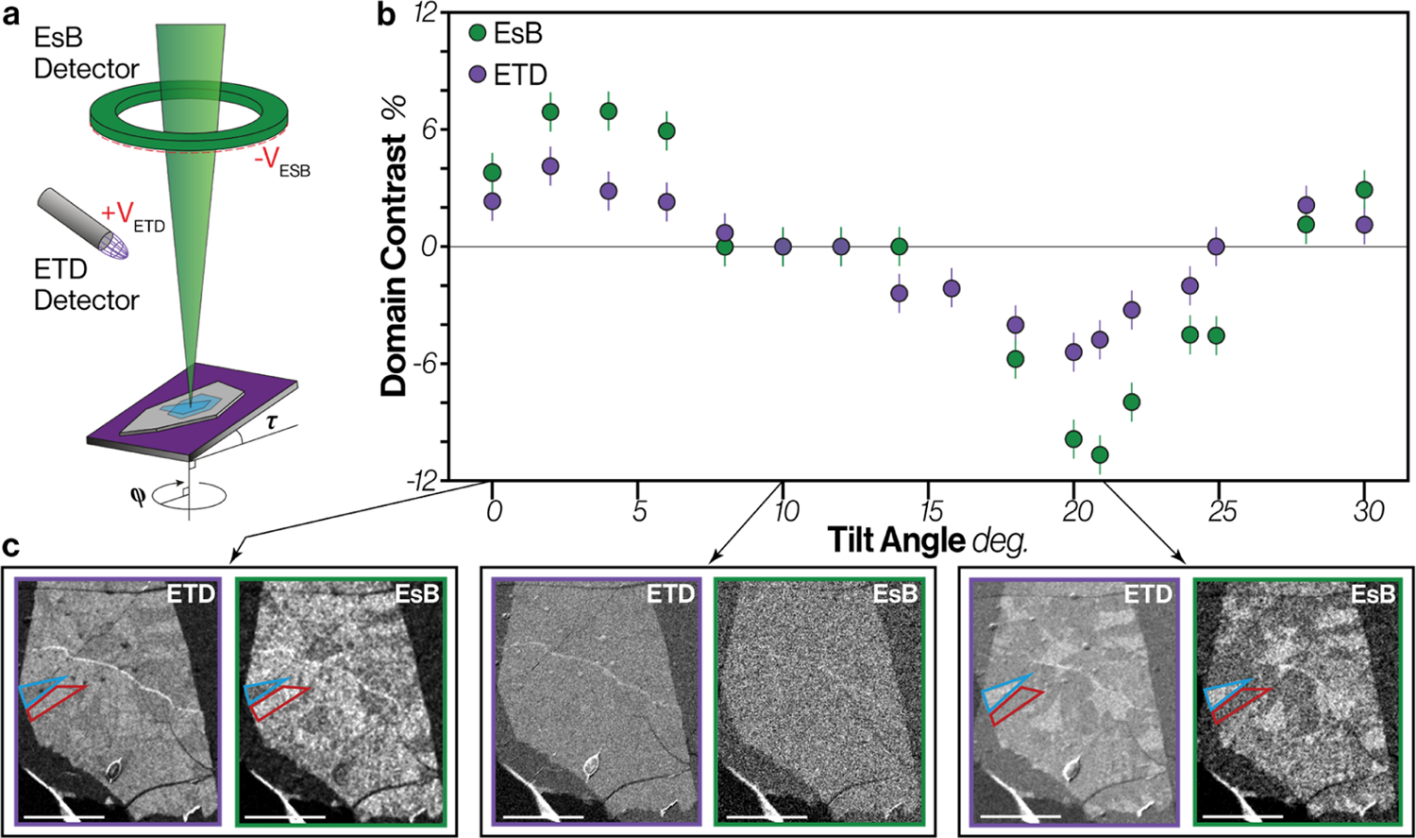
Domain contrast as a function of tilt angle for a 3R type MoS_2_ bilayer on graphite and an oxidized silicon wafer. (a) Schematic diagram with tilt (τ) and rotation (Φ) angles shown. (b) Comparison of domain contrast as a function of specimen tilt angle for the ETD (SE) and EsB (BSE) detectors. Vertical error bars represent the estimated experimental error. (c) The ETD and EsB image pairs for stage tilt angles of 0° (positive channeling contrast), 10° (no contrast) and 22° (negative channeling contrast). Tilted images have been corrected for tilt distortion. The EsB image here is noisier than the ETD image as a result of the use of a larger working distance which facilitates large tilt angles but decreases the collection efficiency of the EsB detector relative to the EsB (see SI Figure S4.1a,b for detail). All scale bars are 3 μm.

In results presented to this point, we have demonstrated the significant domain contrast that is obtained for TMDs in ETD and EsB images even without specimen tilting. Yet previous work on channeling contrast imaging of 2D stacking domains relied on precise specimen tilting and azimuthal alignment with the ETD detector position to achieve contrast.^3,35^Figure 4 shows that indeed tilting the sample has a strong effect on both the EsB and ETD domain contrast for twisted MoS_2_ bilayers, and correct tilt conditions can be used to improve contrast. Channeling contrast is expected to vary with an azimuthal angle^3,34^ (see calculations in SI Figure S3.16). We find that a tilt angle, τ, of ∼21° gives the greatest domain contrast although compared to 0° there is a contrast is reversal at ∼11° (Figure 4b). To determine the optimal azimuthal direction for specimen tilt, the tilted sample was rotated until optimal contrast is achieved (see SI Figure S4.8). For EsB images it is only necessary to tilt along a specific crystallographic azimuthal direction, but for ETD imaging this crystallographic direction must be aligned toward the position of the ETD in the SEM chamber to prevent detector shadowing. Supporting Video 1 shows the domain contrast inversion as a function of sample tilt and SI Figure S4.5 shows a similar effect of specimen tilt on domain contrast from WS_2_. The observation that ETD and EsB signals oscillate in phase supports our conclusion that the main contributor to the ETD domain contrast is the low energy electrons produced by the higher energy channeling signal. The strongest domain contrast observed here was from the EsB detector, which for an MoS_2_ twisted bilayer reaches −11% at the optimal tilt of 21°. The SEM image contrast for 2D materials can be changed by many things other than stacking variations, such as changes in thickness (holes or islands) and or contamination. Twist domains are also often irregular in shape as shown in Figures 1a, 3a,b and 4c. Consequently, knowledge of the oscillation in domain contrast for dynamic tilting makes this a useful tool to effectively remove any ambiguity regarding the presence of atomically reconstructed domains.

### Twist Domain Contrast with Encapsulation

**Figure 5:**
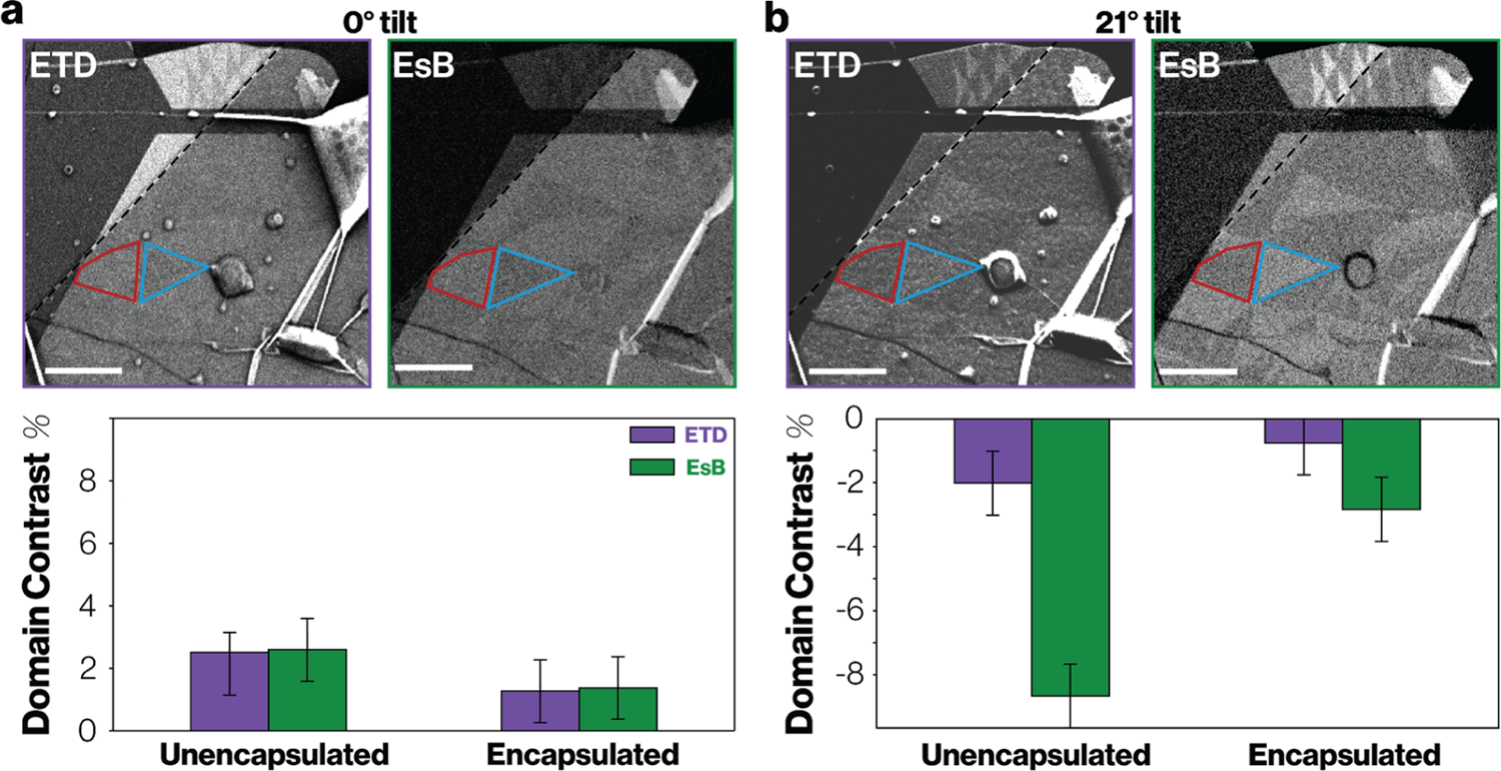
Change in domain contrast with and without 10-layer hBN encapsulation at (a) 0° and (b) 21° specimen tilt, respectively. The top row consists of ETD and EsB images of moiré superlattice domains in twisted bilayer MoS_2_ where a 3.5 nm hBN encapsulation layer covers only the lower part of the specimen. The edge of the hBN flake is indicated by the black/white dashed line, hBN thickness of 3.5 nm was determined via atomic force microscopy. Scale bars are 1 μm. Bottom row shows the quantified mean contrast between domains (note the inversion of domains between 0 and 21° specimen tilt consistent with the data in Figure 4). Error bars represent the variation of the measured contrast for different domains within the image, as described in SI Section 2.

We now consider the applicability of the BSE ECCI approach for imaging stacking domains in realistic electronic device architectures which require encapsulation, capping layers or top-gates. Previous work has shown that ETD images can show stacking differences in twisted WSe_2_ bilayer encapsulated in 5 nm of graphene/hBN, but it was necessary to tilt to 40° and use an electron beam energy of 3000 eV.^35^ Furthermore, no quantification of the domain contrast degradation due to encapsulation was presented. Here we quantitatively compare the change in domain contrast after encapsulation by fabricating a twisted MoS_2_ bilayer with a 3.5 nm thick (10 layer) hBN encapsulation layer that only covers part of the sample: the edge is indicated by the dashed lines in Figure 5a,b. For both the ETD and EsB images at zero tilt, hBN encapsulation decreases the positive contrast by approximately 50% compared to the unencapsulated case (Figure 5a). At ∼21° tilt, the negative domain contrast is reduced by approximately 65% for both signals (Figure 5b), where the further reduction relative to that seen at zero tilt can be explained by the increased thickness of the hBN seen when the sample is tilted. The observation that both ETD and EsB signals are reduced by the same amount again supports our conclusion that the ETD signal originates from channeled higher energy electrons; if the signal originated from channeling of low energy electrons they would not escape from the encapsulation layer (since SE with energies of less than 50 eV have an escape depth less than 1 layer of MoS_2_ or less than 1 nm hBN, see SI Figure S3.9).^44^ These results provide further evidence that the domain contrast in the ETD signal is generated by the channeling of higher energy electrons backscattered through the bilayer.

Surface contamination will also act to degrade the domain contrast compared to perfectly clean surfaces. The extent of this contamination induced degradation, resulting from polymer transfer layers or electron beam deposited hydrocarbon layers, can be expected to be similar to that quantified for hBN for equivalent thicknesses, as both types of contamination have similar mean atomic number to hBN. We have found that *in situ* plasma cleaning is able to reduce the deposition of surface contamination during serial SEM imaging and can even facilitate the cleaning of a contaminated surface by the electron beam resulting in improved contrast for twist domains (see SI Figure S4.9 and Supporting Video S2).

### Twist Domain Contrast for Thicker Samples

**Figure 6:**
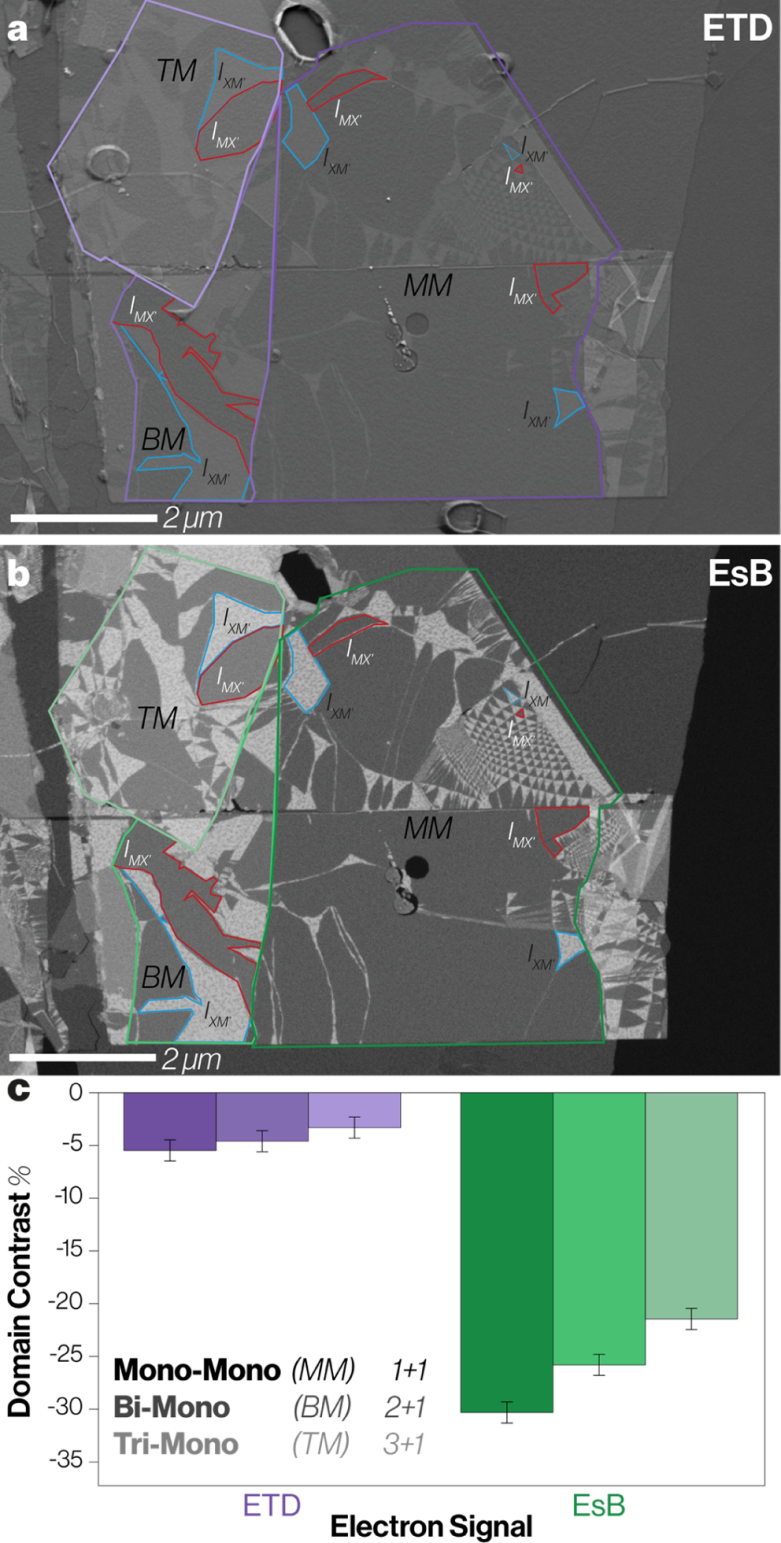
Domain contrast from (a) ETD and (b) EsB images for twisted WS_2_ heterostructure regions including TMD layers with different thicknesses (sample is unencapsulated). Different thicknesses of the lower TMD layer are indicated by the outlines in (a) and (b). The light-to-dark outlines highlight monolayer-trilayer (TM), monolayer–bilayer (BM), and monolayer-monolayer (MM) domain thicknesses, respectively. For each region, the specific domains used for measuring *I*_XM_ and *I*_MX_ values are shown via the red and blue polygons, respectively. (c) The twist domain contrast as a function of bottom TMD layer thickness. Imaging was performed with a working distance of 5.5 mm, an acceleration voltage of 1.5 kV, and a stage tilt of 20.2°.

Figure 6 compares the effect on twist domain contrast when the lower TMD layer is thicker (a monolayer on a pristine bilayer or monolayer on a pristine trilayer). These heterostructures have reduced domain contrast compared to the bilayer consisting of a monolayer on a monolayer. We assign this to the increased thickness of the lower layer adding to the background signal without adding to the channeling difference between the domains (Figure 6c). Nonetheless, we find that the EsB domain contrast signal for twist domains in WS_2_ remains high (above 20%), even when the lower WS_2_ crystal is 3 layers thick. For this sample/imaging configuration the EsB signal gives greater domain contrast than the ETD signal for all heterostructures, but the additional thickness of the lower layers decreases the observed contrast of both detectors by a similar factor, further supporting our hypothesis that the ETD signal is coupled to the relative populations of higher energy electrons emerging from the sample.

## Conclusions

This work demonstrates the largely unexploited potential of conventional SEM imaging for revealing reconstructed twist domain networks in TMD heterostructures. We find that channeling contrast SEM imaging of twisted domains is achievable for most SEM models and configurations (examples shown in SI Figure S4.10), without requiring prior knowledge of sample crystallinity, supporting the widespread opportunities for applying the ECCI approach for imaging stacking domains in TMDs.

Our calculations demonstrate that for transmitted electrons in suspended bilayers, domain contrast inversion occurs at moderate scattering angles, with positive contrast from inelastic electrons dominating at high angles and negative contrast from elastic interactions (mainly the {1̅100} Bragg reflection) important at low angles. Optimal domain contrast for transmitted electrons at 1500 V accelerating voltage and zero specimen tilt is thus achieved by choosing an annular STEM detector with either (i) a large collection angle and an inner detector angle of ∼25*°* to exploit the positive contrast from inelastically scattered electrons or (ii) a detector that has a small angular range to exclude the directly transmitted beam and the electrons from high-angle scattering and a collection angle centered on the {1̅100} Bragg reflection to achieve negative contrast. The EsB detector also gives strong positive domain contrast by collecting the reflected inelastic BSEs from suspended samples.

Both MoS_2_ and WS_2_ bilayers on conventional graphite/SiO_2_ substrates give SEM images with positive twist domain contrast even without specimen tilt (when imaging along the [0001] direction) for all detectors (EsB and InL and ETD). Our calculations suggest that the positive domain contrast seen on the EsB detector for the suspended sample is enhanced by the presence of the substrate, which adds positive contrast contributions from both elastic and inelastic scattering contributions. The situation is more complicated for the ETD signal where the weak negative contrast observed for suspended samples is overwhelmed and the ETD detector signal for supported samples is due to low energy secondary electrons generated by the higher energy electron signals. Thus, the domain contrast of TMDs on bulk substrates always behaves in a similar way whether high or low energy electrons are being detected.

A specimen tilt of 21° away from the [0001] direction can be applied to increase domain contrast by ∼4 times relative to the 0° case (accompanied by an inversion in domain contrast). WS_2_ bilayers have approximately double the contrast of MoS_2_ resulting in twist domain contrasts of −31 and −16% for WS_2_ and MoS_2_, respectively, at the optimal tilt angle of 21°. Importantly for practical use to image electronic structures, the twist domains are visible for both WS_2_ and MoS_2_ bilayers beneath a 3.5 nm hBN encapsulation layer, in the presence of hydrocarbon surface contamination, and for complex heterostructures including trilayer TMDs. Although imaging twist domains with the conventional ETD SE imaging mode is entirely possible, we find the EsB, InL and other annular BSE detectors are generally preferrable for several reasons. First, annular BSE detectors generally allow smaller working distances and therefore higher spatial resolution to be achieved than is possible for ETD detectors. Second, the optimal sample tilt does not need to be toward the ETD detector. Third, the higher energy electron signal is less sensitive to surface topography and charging so is also capable of revealing domain contrast where the low energy signal fails, such as where there are encapsulation bubbles or for insulating heterostructures.

We believe this readily accessible and inexpensive approach to image twisted domain configurations in fabricated (opto)electronic devices has great potential to improve understanding of how local variations in twist angle and lattice strain effect optimal device performance, as well as enabling *in situ* SEM measurements of moiré superlattices and variable stacking configurations in TMDs.

## Methods

### Sample Preparation

Samples were prepared from mechanically exfoliated crystals via a modified tear-and-stack method.^38^ Specific details can be found in SI Section 1. The use of metal-coated substrates or lithographic electronic contacts is recommended as charging becomes a significant obstacle to the visualization of twisted 2D materials with SEM, as illustrated in SI Figure 2.7.

### Scanning Electron Microscopy

The primary SEM used for this investigation was the Zeiss Merlin Gemini II. An acceleration voltage of 1500 V, beam current of 1 nA, a pixel dwell time of 52 μs, working distance of 4–5 mm, and a 400 and −800 V detector bias for the ETD and EsB detectors, respectively, were used when modulating channeling contrast with stage parameters. Detector annular collection ranges are 179–160° degrees for the EsB, 160–150° for the InL (angles measured from the primary beam forward direction) for a primary beam energy of 1500 eV. The STEM detector angular range varies with detector-sample distance, in the range of 1.2–16.0° for STEM2 and 25.6–55.0° for STEM5 (angles measured from the directly transmitted beam for a detector-sample distances of 5.8–13.0 and 5.7–11 mm, respectively). The scanning transmission electron microscopy (STEM) data collected with the Merlin’s segmented STEM detector. Other microscopes used include an FEI Magellan, Apreo SEM, Helios 660 FIB SEM and a Zeiss Ultra with similar microscope parameters as listed above.

## Supporting Information Available

Information on 2D heterostructure sample preparation, methodology for quantification of domain contrast, methodology for modeling elastic and inelastic contributions to electron scattering, effect of SEM experimental conditions on domain contrast including induced carbon contamination (PDF)
Video S1: Top ETD and bottom EsB electron image tilt series which has been aligned to minimise tiltdistortion and drift to highlight changes in domain contrast (MP4)
Video S2: Top ETD and bottom EsB electron image time series showing contrast improvement during SEM imaging (MP4)
