# Supplementary material for: Scanning Electron Microscopy Imaging of Twist Domains in Transition Metal Dichalcogenide Heterostructures
Source: ACS Nano. 2024 Dec 6;18(50):34023–33. doi: 10.1021/acsnano.4c09364 (PMC11656840; doi:10.1021/acsnano.4c09364)
Supplement: Supplementary file 1 — nn4c09364_si_001.pdf [file nn4c09364_si_001.pdf]

Supplementary Information for:

Scanning Electron Microscope Imaging of Twist Domains in Transition Metal Dichalcogenide Heterostructures

Evan Tillotson[1,3], James G. McHugh[2,3], James Howarth[3], Teruo Hashimoto[1], Nicholas J. Clark[1,3], Astrid Weston[2,3], Vladimir Enaldiev[2,3], Sam Sullivan-Allsop[1,3], William Thornley[1,3], Wendong Wang[2,3], Matthew Lindley[1], Andrew J. Pollard[4], Vladimir I. Fal'ko[2,3^], Roman V. Gorbachev[2,3#] and Sarah J. Haigh[1,3].*

1. Department of Materials, University of Manchester, Manchester M13 9PL, UK
2. Department of Physics and Astronomy, University of Manchester, Manchester M13 9PL, UK
3. National Graphene Institute, University of Manchester, Manchester M13 9PL, UK
4. National Physical Laboratory, Hampton Rd, Teddington, TW11 0LW, UK

sarah.haigh@manchester.ac.uk *; roman@manchester.ac.uk #; falko@manchester.ac.uk ^.

Contents

1. Sample Preparation 2

2. Quantification of Domain Contrast 2

3. Modelling of Electron Scattering as a Function of Collection Angle 10

3.1. Elastic Scattering 10

3.2. Inelastic Scattering 18

3.2.1. Inelastic loss processes 18

3.2.2. Inelastic scattering mean-free paths 19

3.2.3. Monte Carlo modelling 20

3.2.4 Angular dependence 26

4. Optimising SEM Instrument Parameters 27

4.1 Domain contrast comparisons with varying SEM parameters 27

4.2 Effect of beam induced carbon contamination on domain contrast 32

4.3 Imaging MoS2 domain contrast in different SEMs 34

Caption for Supplementary Video S1 35

Caption for Supplementary Video S2

References 36

1. Sample Preparation

A modified version of the tear-and-stack technique[1] was used to prepare the 3R-type twisted TMD samples on a Si/SiO_2 wafer substrate, using a micromanipulation transfer rig in an argon atmosphere glove box. The monolayer and few-layer TMD flakes were prepared by mechanically exfoliating bulk crystals onto Si/SiO_2 wafers spin coated with polypropylene carbonate (PPC). The micromanipulator needle was employed to tear the exfoliated flake in half, so that a poly-methyl methacrylate (PMMA) carrier layer could be deposited to selectively pick up one half of a flake (at 55 °C) and deposit it on to the second half with a specified rotation. The 3R-type homo-bilayers were then either deposited onto a graphite crystal on a Pt (50 nm)/Ti (5 nm) coated Si/SiO_2 wafer, or on to a thick hBN crystal. To achieve hBN-encapsulation, a further hBN crystal (2-3.5 nm thick as determined by AFM) was then transferred using a PMMA carrier layer. In the absence of the metallic Pt-Ti layer, the bilayer was grounded *via* lithographically patterned electrodes. The whole 2D van der Waals heterostructure was then annealed in a vacuum chamber (pressure approximately 10^{-6} mbar) at 200°C to minimise surface contamination prior to SEM imaging. To produce the freely suspended heterostructure samples the twisted bilayers were picked up using a patterned hBN crystal and transferred to a holey SiN_x support.

2. Quantification of Domain Contrast

Here we employ Michelson contrast, C_m to compare domain contrast for different conditions:

$$C_m = 100\,\frac{I_d}{I_s} = 100\,\frac{I_2 - I_1}{I_2 + I_1}$$

Where I_1 and I_2 are the mean intensity of the two stacking domains, I_d is the intensity difference (I_1 - I_2) and I_s is the intensity sum (I_1 + I_2). The mean intensity value for a specific domain region, I_1 or I_2, was determined by extracting pixel by pixel intensity histograms for a single domain using the ImageJ software. These histograms also allowed measurement of the standard deviation in the individual pixel intensities for the individual domain (ΔI_1 and ΔI_2). To determine the measurement errors within the selected domains the reported contrast values $\Delta C_{m,}$ standard deviation errors have been propagated in accordance with standard theory:

$$\Delta C_m = \sqrt{\left(\frac{\partial c_m}{\partial I_1}\Delta I_1\right)^2 + \left(\frac{\partial c_m}{\partial I_2}\Delta I_2\right)^2} \qquad \therefore \qquad \Delta C_m = \sqrt{\left(\frac{200\Delta I_1 I_2}{(I_1+I_2)^2}\right)^2 + \left(\frac{200 I_1 \Delta I_2}{(I_1+I_2)^2}\right)^2}$$

However, the human error of selecting domains to analyse was found to be larger than this in most cases and is estimated at ±1%. The data is therefore reported with whichever of these errors is larger. **Figure S2.1** provides an overview of the method used to measure individual domain contrast for an MoS_2 bilayer region (A1). First the domain region in the image is isolated. Any contamination bubbles or defects present (see **Figure 1a**) are excluded from the region of interest (see **Figure S2.1c**) so they do not contribute to the intensity histogram. Selecting the pixels that contribute to a single domain enables measurement of the intensity histogram whose mean intensity value and standard deviation provide the necessary values for I_1 and ΔI_1, respectively. The process is repeated for a second domain with the different contrast as near as possible to the first domain to measure I_2 and ΔI_2, enabling C_m and ΔC_m to be determined.

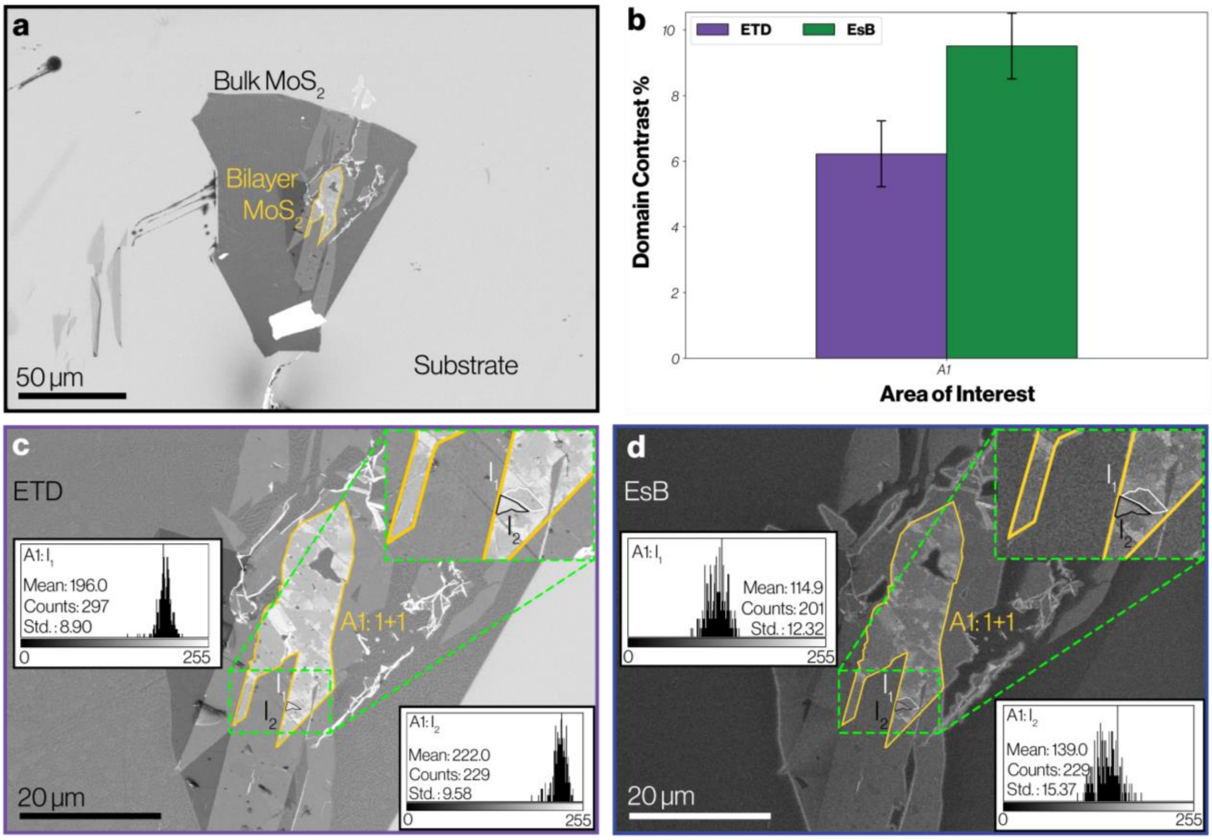

Figure S2.1: Method of measuring the Michelson contrast of reconstructed twist domains in an MoS_2 twisted bilayer (not encapsulated). a) An ETD SEM overview image with highlighting of the bilayer of MoS_2 (outlined in yellow) within the bulk flake. **b**) Plot of the Michelson contrast for the twisted domains measured from ETD and EsB images in area A1. Imaging parameters: 5.9 mm working distance, 1.0 kV acceleration voltage, 0.1° stage tilt. **c**) & **d**) ETD and EsB images of the region containing the twist domains highlighted by the yellow boundary in panel **a**). The insets (green border) in the top right show a magnified region where the individual domains are outlined in white and black, having intensities of I_1 and I_2 respectively. Also inset are intensity histograms from the individual domains (I_1 and I_2) with the mean and standard deviation (std.) values, along with the number of counts (pixels) shown. Often the separate domain intensities were not distinguishable in the intensity histogram of the full area (yellow polygon) so the evaluation of bright/dark domain intensities and their standard deviations was performed by measurements made from individual domains using ImageJ.[2]

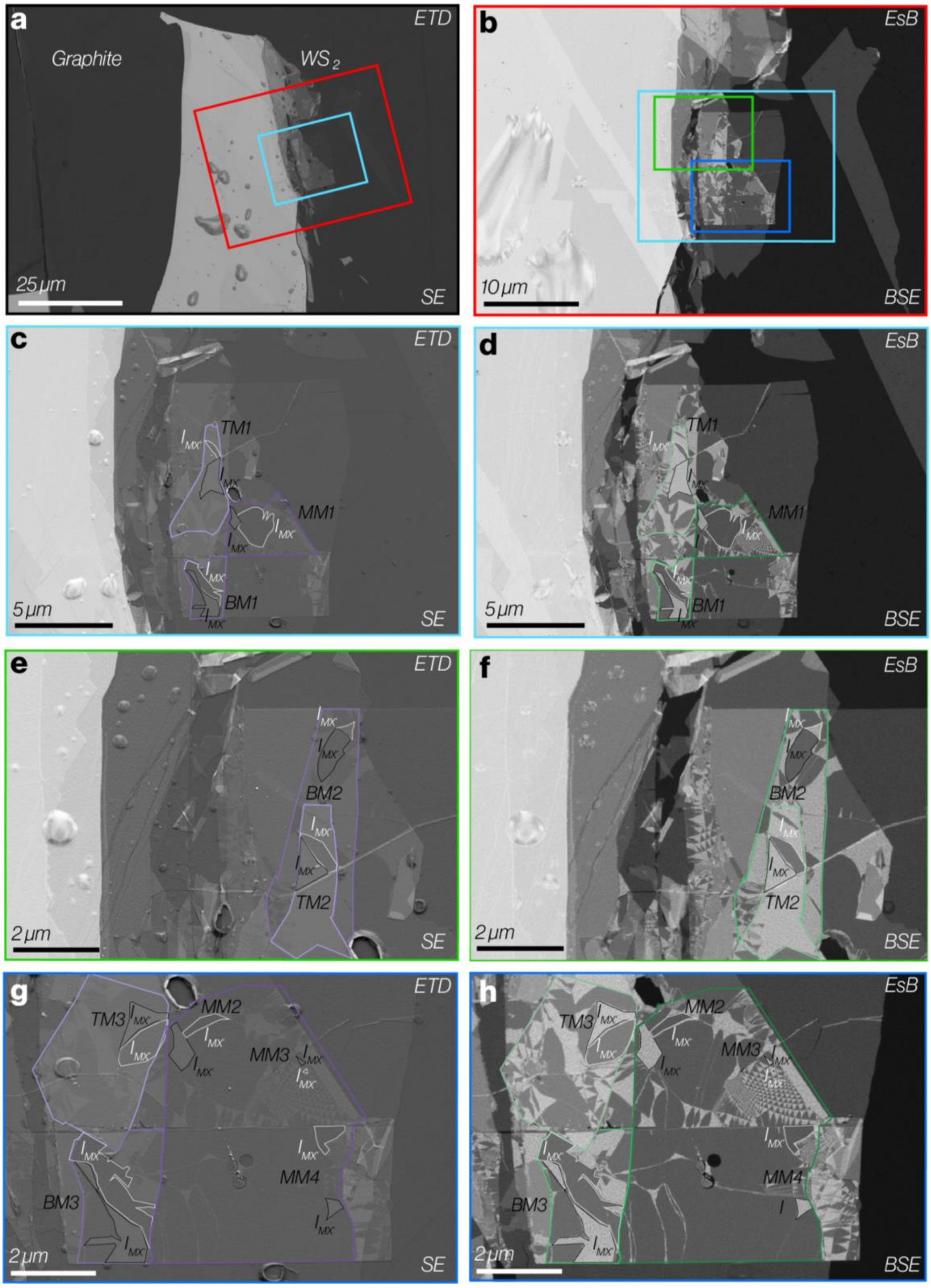

Figure S2.2: Comparison of domain contrast from a twisted WS_2 heterostructure including different thicknesses for the bottom layer (not encapsulated). Left column, **a**, **c**, **e** and **g**, are ETD images. Right column, **b**, **d**, **f** and **h**, are EsB images. Different thickness regions are indicated *via* differing shades of coloured outlines in purple and green for ETD and EsB images, respectively. The lightest shading is monolayer on trilayer (TM). The medium shading corresponds with monolayer on bilayer (BM) regions while the darkest shading highlights monolayer on monolayer (MM) areas. For each region, the specific domains used for measuring I_1 and I_2 values are shown *via* the black and white polygons, respectively. The intensity histograms of these domains can be found in **Figure S2.3**. Imaging parameters 5.5 mm working distance, 1.5 kV acceleration voltage, 20.2° stage tilt.

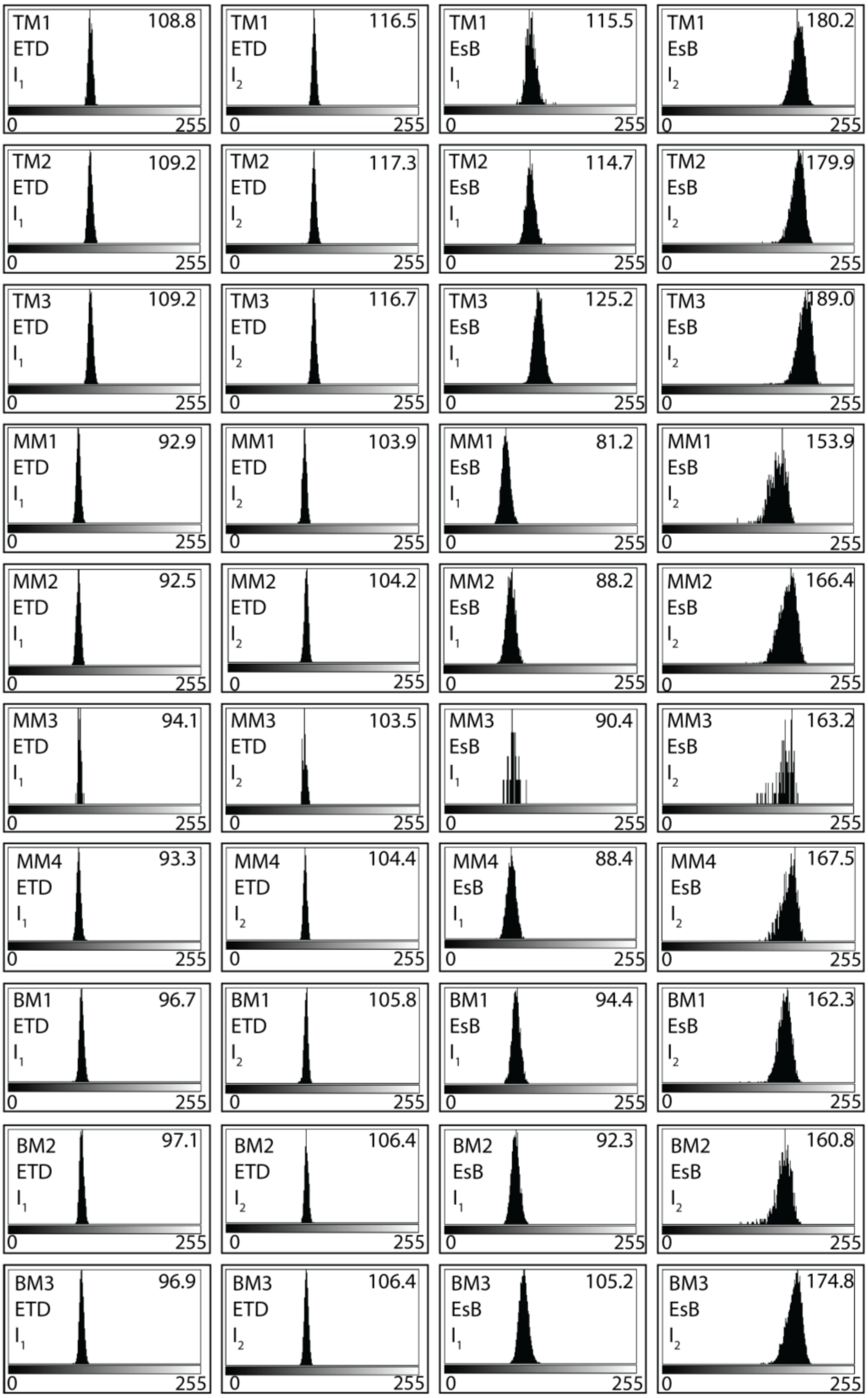

Figure S2.3: Intensity histograms measured from the WS_2 domains outlined in Figure S2.2. All images were analysed as 8-bit TIFs (0-255). Mean intensity values are given top right for each histogram.

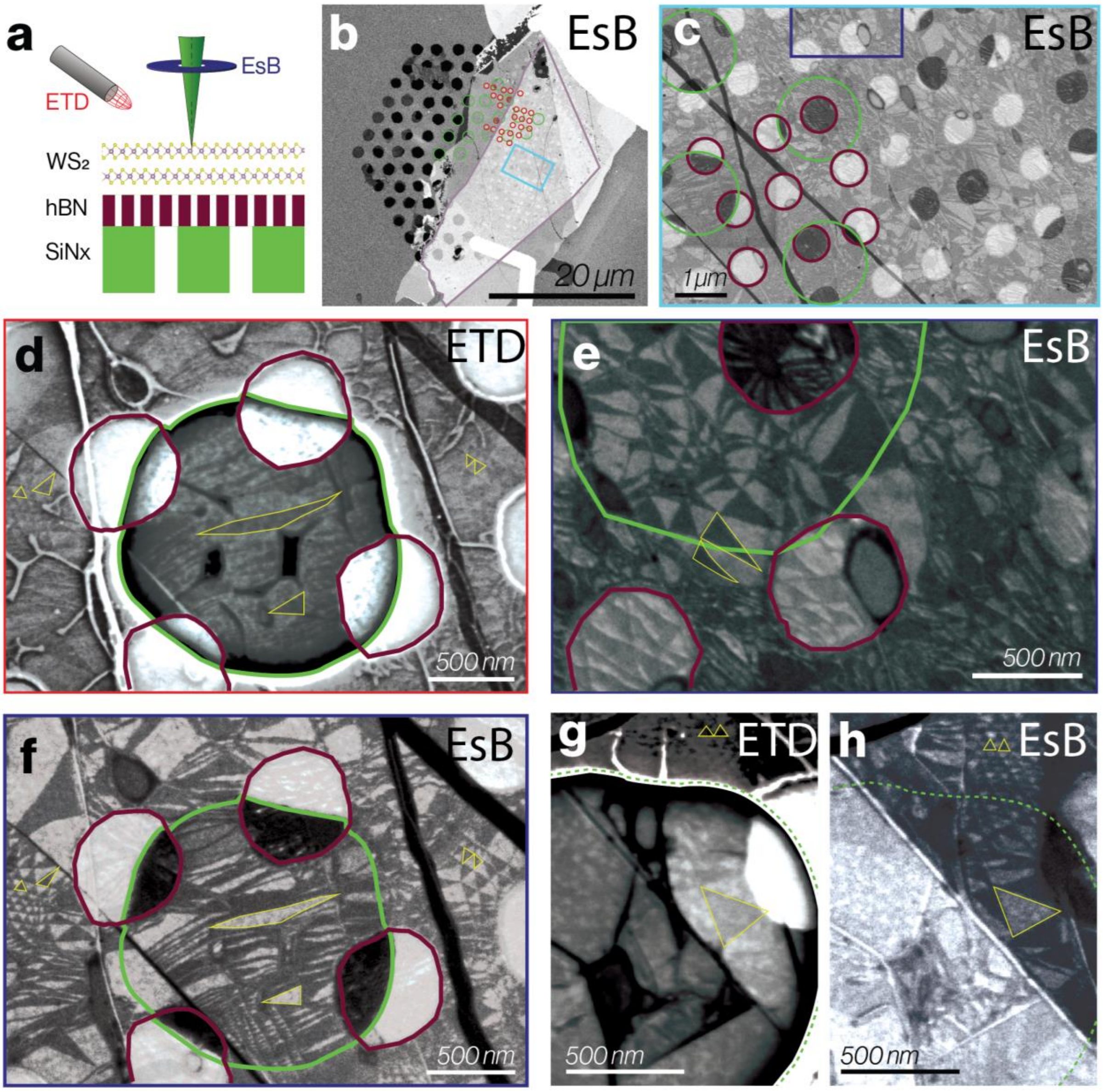

Figure S2.4: Comparison of ETD (red) and EsB (blue) electron detector signals for a hBN supported WS_2 bilayer. a) Schematic cross-section of the suspended WS_2 bilayer supported on a hBN substrate with 500 nm diameter holes (red borders) and a SiN_x TEM grid with 2μm diameter holes (green borders). **b**) and **c**), EsB images of double-suspended twisted WS_2 bilayer at low and high magnifications, respectively, where **c**) corresponds with the blue rectangle highlighted in **b**). **d**) & **g**) ETD images and **f**) & **h**) EsB images showing contrast inversion within pseudo-freely-suspended regions when comparing SE and BSE images. **e**) BSE image showing retention of contrast from freely-suspended to substrate-supported areas.

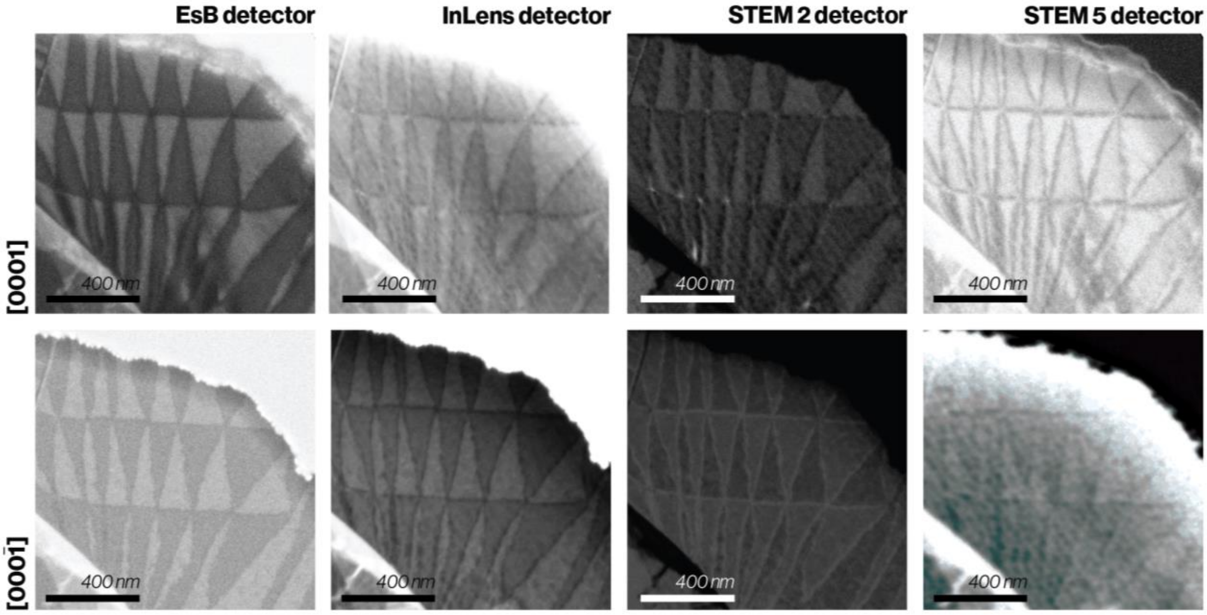

Figure S2.5: Contrast inversion achieved by turning the sample upside down. Top row: SiNx holey support side down. Bottom row: SiN_x holey support side up. Identical samples are viewed along close to either the [0001] (top) or [000$\bar{1}$] (bottom) crystallographic direction. Identical detector conditions are used for both orientations. Contrast for STEM5 detector is reduced in the [000$\bar{1}$] images, likely due to surface contamination. The magnitude of this domain wall contrast has been quantified as 3.7%, 5.5%, 8.4% and 4.6% for the EsB, InLens, STEM2 and STEM5, detectors respectively (the Michelson contrast has been measured relative to the local domain with the closest intensity and averaged across both rows of images in **Figure S2.5**)

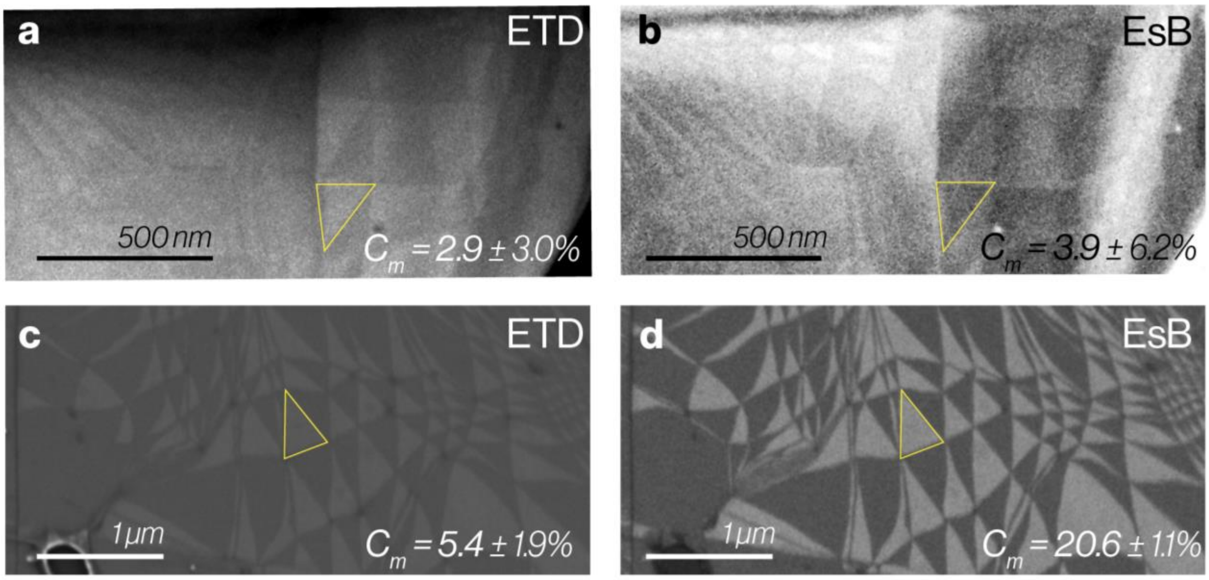

Figure S2.6: Comparison of ETD and EsB signals for suspended and supported WS_2 twisted bilayers. a) & **b**) are suspended samples, indicating contrast inversion between the two imaging modes. **c**) & **d**) are twisted bilayers on a solid graphite/SiO_2 substrate the two signals have qualitatively similar domain contrast.

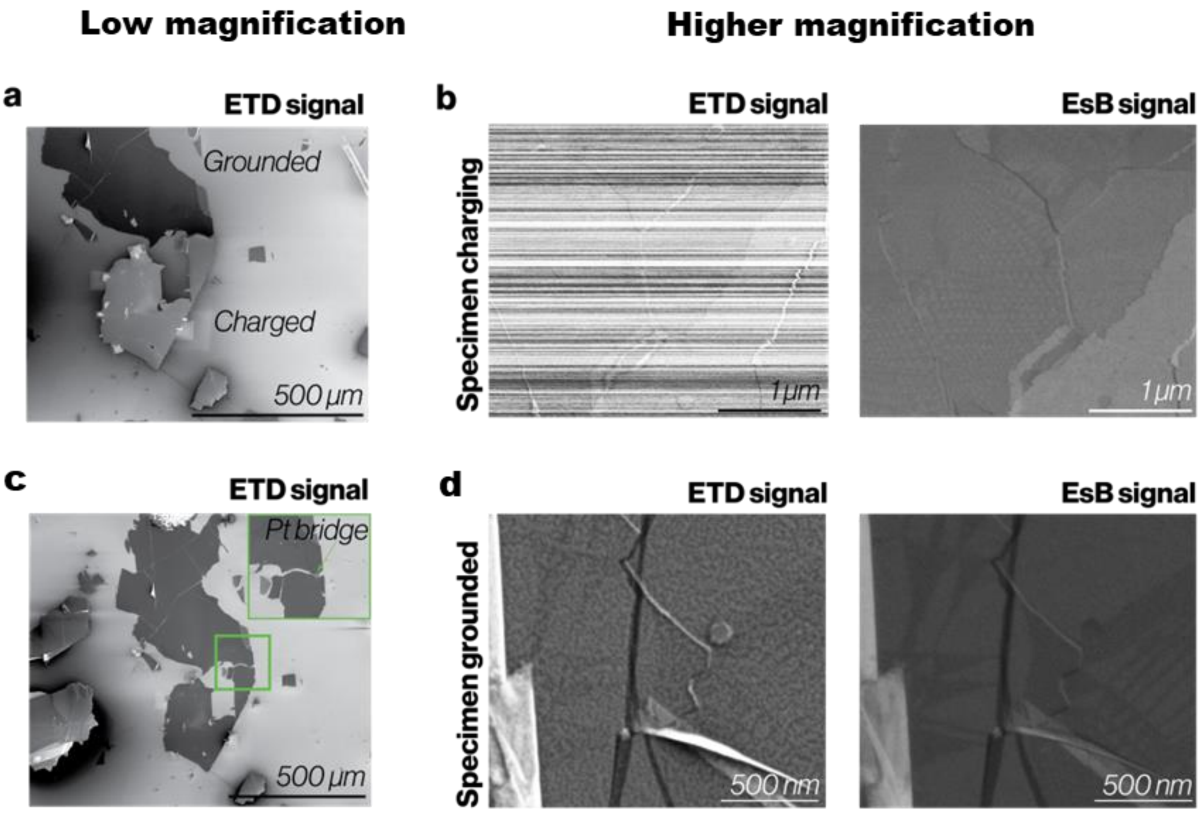

Figure S2.7: Effect of specimen charging on ETD and BSE images. a) Shows the sample as initially fabricated, where the flake towards the bottom of the image is charging (different contrast observed between the flakes. **b**) Higher magnification imaging of this charging flake is possible with the EsB detector but not with the ETD. **c**) Shows the same region as in a) after the bottom flake has been grounded by an electron beam deposited platinum strip. The inset shows an enlarged view of the Pt bridge. **d**) After the Pt strip deposition both ETD and EsB signals are restored. However, the topography caused by unintended Pt deposition outside the region of the Pt strap obscures the visibility of the channelling contrast in the ETD signal.

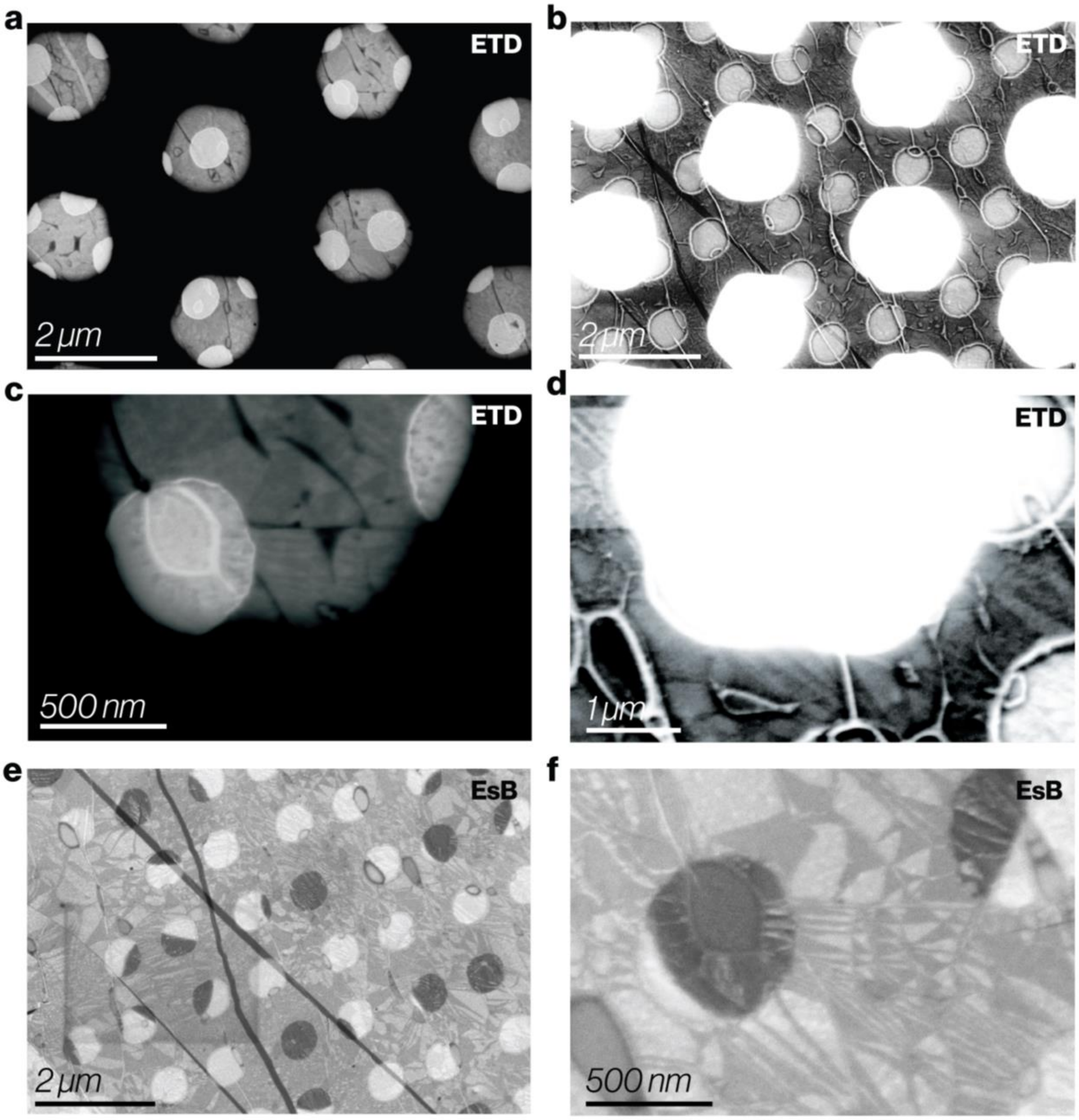

Figure S2.8: Raw images (c & d) of the region shown in Figure 3a (main text). a) & **b**) and **c**) & **d**), Low and high magnification ETD images, respectively, demonstrating two images which have been stitched together due to insufficient dynamic range, such as **Figure 3a** in the main text. **e**) & **f**), EsB images corresponding with **a**) & **c**), respectively, evidencing sufficient dynamic range to image substrate-supported and freely-suspended regions simultaneously.

3. Modelling of Electron Scattering as a Function of Collection Angle

3.1. Elastic Scattering

The total electron scattering contains both elastic and inelastic scattering processes which need to be considered differently (see **Figure S3.1**).

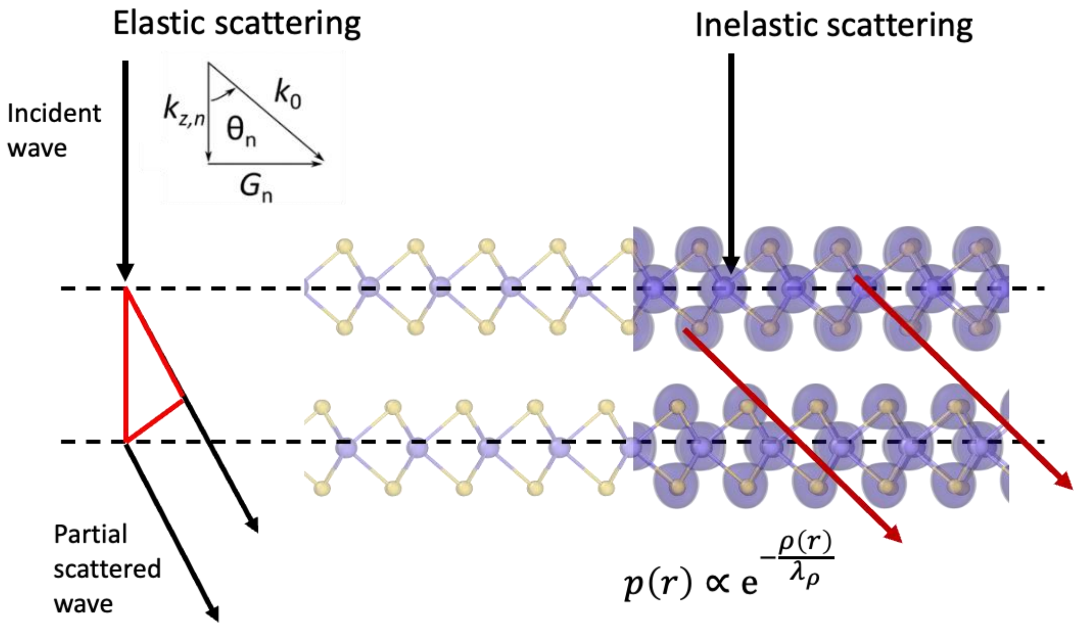

Figure S3.1: Elastic vs inelastic processes. We define transmission of the electrons when the scattering angle satisfies the condition $\theta < 90^o$ and reflection of the electrons when $\theta > 90^o$.

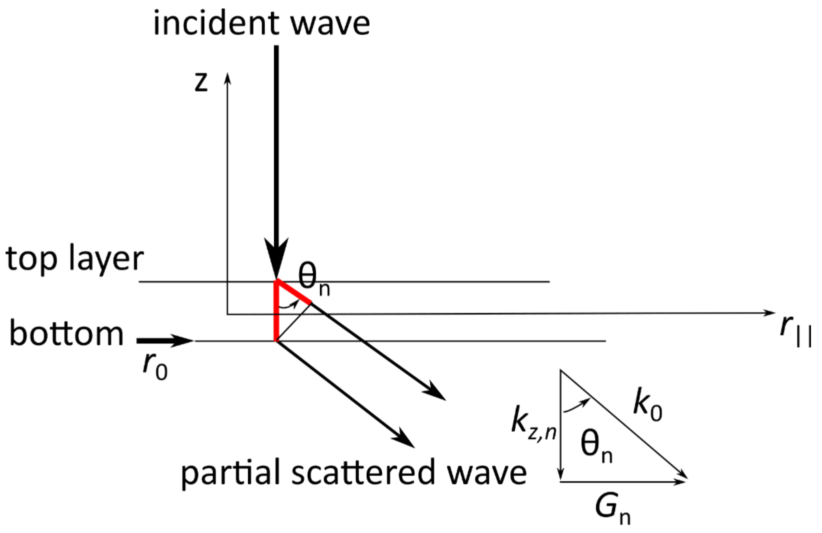

Figure S3.2: Geometry of elastic scattering occurring within the two layers of the TMD.

First, we consider modelling of the elastic scattering behaviour, the geometry of which is illustrated in **Figure S3. 2**. Energy conservation for scattering gives:

$$k_0^2 = k_{z,n}^2 + G_n^2 \text{ ; } (\mathbf{1}),$$

where k_0 is the wave vector of incident electrons, and G_n is a reciprocal vector of the TMD bilayers (not necessarily the basic reciprocal vector). The direction of the partially scattered electron wave (G_n) is given by the angle:

$$\theta_n = \arcsin\left(\frac{G_n}{k_0}\right); \ (\mathbf{2}).$$

The partial wave, ψ_n, scattered in the angle θ_n , is given by a sum of waves scattered from the top and bottom TMD layers,

$$\psi_n = Ae^{ik_{z,n}z+i\boldsymbol{G}_n\boldsymbol{r}_{||}} + Ae^{ik_{z,n}z+i\boldsymbol{G}_n(\boldsymbol{r}_{||}-\boldsymbol{r}_0)+i\Delta\varphi_n}; \ (\mathbf{3}).$$

Here, $\Delta\varphi_n = k_0 d(1 - cos(\theta_n)) = d(k_0 - k_{z,n}) = d\left(k_0 - \sqrt{k_0^2 - G_n^2}\right)$ is a phase shift coming from the difference in path lengths (red lines in Figure S3.2), and $\boldsymbol{r}_0$ is offset between lattices of the two layers ($\boldsymbol{r}_0 = \pm(0, a/\sqrt{3})$ for MX' (top sign) XM' (bottom sign) stackings and $\boldsymbol{r}_0 = 0$ for XX stacking).

The intensity of the partial wave reaching a detector is,

$$I_n = |\psi_n|^2 = 2A^2\left(1 + \cos(\boldsymbol{G}_n\boldsymbol{r}_0 - \Delta\varphi_n)\right); \ (\mathbf{4}).$$

The difference in the intensity for the two domains, $I_{MX} - I_{XM}$ and the sum intensity for the two domains, $I_{MX} + I_{XM}$ are therefore given by:

$$I_{MX} - I_{XM} \propto A(E, \theta_n)\sin(\boldsymbol{G}_n\boldsymbol{r}_0^{MX})\sin(\Delta\varphi_n(E)); \ (\mathbf{5}),$$

$$I_{MX} + I_{XM} \propto A(E, \theta_n)[1 + \cos(\boldsymbol{G}_n\boldsymbol{r}_0^{MX})\cos(\Delta\varphi_n(E))]; \ (\mathbf{6}).$$

The n index subscript is related to the 2D reciprocal vector indexes $\{n_1, n_2\}$ by the following equation (see **Figure S3.3**):

$$\boldsymbol{G}_n = n_1\boldsymbol{G}_1 + n_2\boldsymbol{G}_2,$$

$$\boldsymbol{G}_1 = \frac{4\pi}{a\sqrt{3}}\left(-\frac{1}{2}, -\frac{\sqrt{3}}{2}\right), \qquad \boldsymbol{G}_2 = \frac{4\pi}{a\sqrt{3}}(0,1).$$

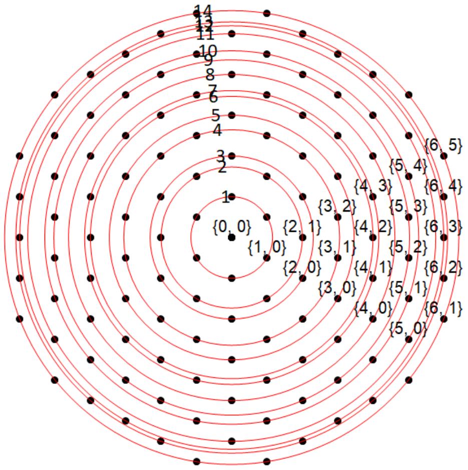

Figure S3.3: Relationship between the n index used in the explanation of elastic scattering and the 2D reciprocal vector indexes where n =1 or {1 0}, is equivalent to the Miller Bravais crystallographic notation of $\{\bar{1}\,1\,0\,0\}$ and where n =2 or {2 1}, is equivalent to the Miller Bravais crystallographic notation of {-$2\,\bar{1}\,1\,0\}$ etc.

The scattering angle θ_n values depend on the accelerating voltage (V) of the primary electron beam as this determines the primary electron energy, E_n. The energy dependent factor is given by:

$$A(E,\theta_n) = \left|\langle k_{z,n}, \boldsymbol{k}_{||} = \boldsymbol{G}_n \middle| V_{\mathrm{Mo}}(\boldsymbol{r}_{||}, z) + V_{\mathrm{S}}(\boldsymbol{r}_{||} + \boldsymbol{\tau}, z + d_{XX}/2) + V_{\mathrm{S}}(\boldsymbol{r}_{||} + \boldsymbol{\tau}, z - d_{XX}/2) \middle| k_0, \boldsymbol{k}_{||} = \boldsymbol{0}\rangle\right|^2; \; \textbf{(7)}.$$

This takes into account angular dependence of the Bragg scattering probability in the Born approximation with the Thomas-Fermi approximation for potentials ($V_{\mathrm{Mo,S}}$) of molybdenum (Mo) and sulphur (S) atoms; $\boldsymbol{\tau} = (0, a/\sqrt{3})$ and where $\pm d_{XX}/2$ are in-plane and out-of-plane distances between Mo and S atoms in the same layer.

The difference in the intensity for the two domains, $I_{MX} - I_{XM}$ and the sum intensity for the two domains, $I_{MX} + I_{XM}$ as a function of E_n are presented in **Figure S3.4a** and **b**, respectively. Here each line, $\theta_n(E) = \arcsin\left(\frac{G_n}{k_0(E)}\right)$, corresponds to a particular value of n characterised by a particular reciprocal vector G_n. Colour maps superimposed on the lines show the magnitude of the intensity contrast between domains and total scattering intensity for **Figure S3.4a** and **b**, respectively.

In **Figure S3.4a**, many scattering angles are seen to give very small or zero intensity for a range of E_n. This happens for two reasons: either (i) $I_{MX} - I_{XM} \propto \sin(\boldsymbol{G}_n \boldsymbol{r}_0^{MX}) = 0$ for given set of 6 reciprocal scattering vectors or (ii) there are angles which contain 12 reciprocal scattering

vectors, for which the two symmetrically equivalent sets of 6 reciprocal vectors give opposite values for $\sin(\boldsymbol{G}_n\boldsymbol{r}_0^{MX})$ leading to zero total contribution.

Figure S3.4c and **Figure S3.4e** show the values of the scattering intensities for a primary electron energy of 1500 eV, providing plots that are similar to those obtained from powder diffraction measurements. **Figure S3.4d** illustrates that at 1500 eV incident beam energy, the significant difference in the intensity of the two domains is for the n=1 ($\{\bar{1}\ 1\ 0\ 0\}$ Miller Bravais) type reflections.

A closer inspection of the energy dependence of G_n reflections in **Figure S3.4a**, reveals that it is possible to selectively choose the collection angle β in order to achieve a reversal of domain contrast, $I_{XM} - I_{MX}$ for primary electron energies of >2000eV and at ~1000eV because for these energies, values of $I_{XM} - I_{MX}$ are positive for at least one G_n and negative for other G_n. Contrast reversal is achieved by selecting the appropriate G_n as exemplified in **Figure S3.5**. However, for the energy interval of 900 – 1500 eV, $I_{XM} - I_{MX}$ is negative for all G_n reflections, so it is impossible to invert the contrast between domains by changing collection angle, when considering just elastic scattering. **Figure S3. 6** shows the elastic contribution to the Michelson contrast $(I_{XM} - I_{MX}\ /\ I_{MX} + I_{XM})$ of the domains for different ranges of collection angle (β) at 1500 eV. This simply shows that to achieve high contrast, it is necessary to choose the angular range to include the scattering from the n=1 $\{\bar{1}\ 1\ 0\ 0\}$ reflections.

A similar consideration of the behaviour of $I_{MX} - I_{XM}$ as a function of E_n shows that when summing elastic scattering contributions over a large angular range the domain contrast will be highest in the interval between 1200 – 1700 eV, since here all contributions to $I_{MX} - I_{XM}$ have the same sign.

Elastic scattering also occurs at large scattering angles with angles 90°>θ>180° corresponding to reflected electrons. As illustrated in **Figures S3.7** and **S3.8**, for these large scattering angles there are many more G_n reflections which contribute to the contrast and they are more sensitive to the precise electron beam energy. However, the intensity of these high angle elastically scattered electron is four orders of magnitude smaller than the transmitted electrons and therefore the elastic signal reflected in the bilayer is considered negligible in this work.

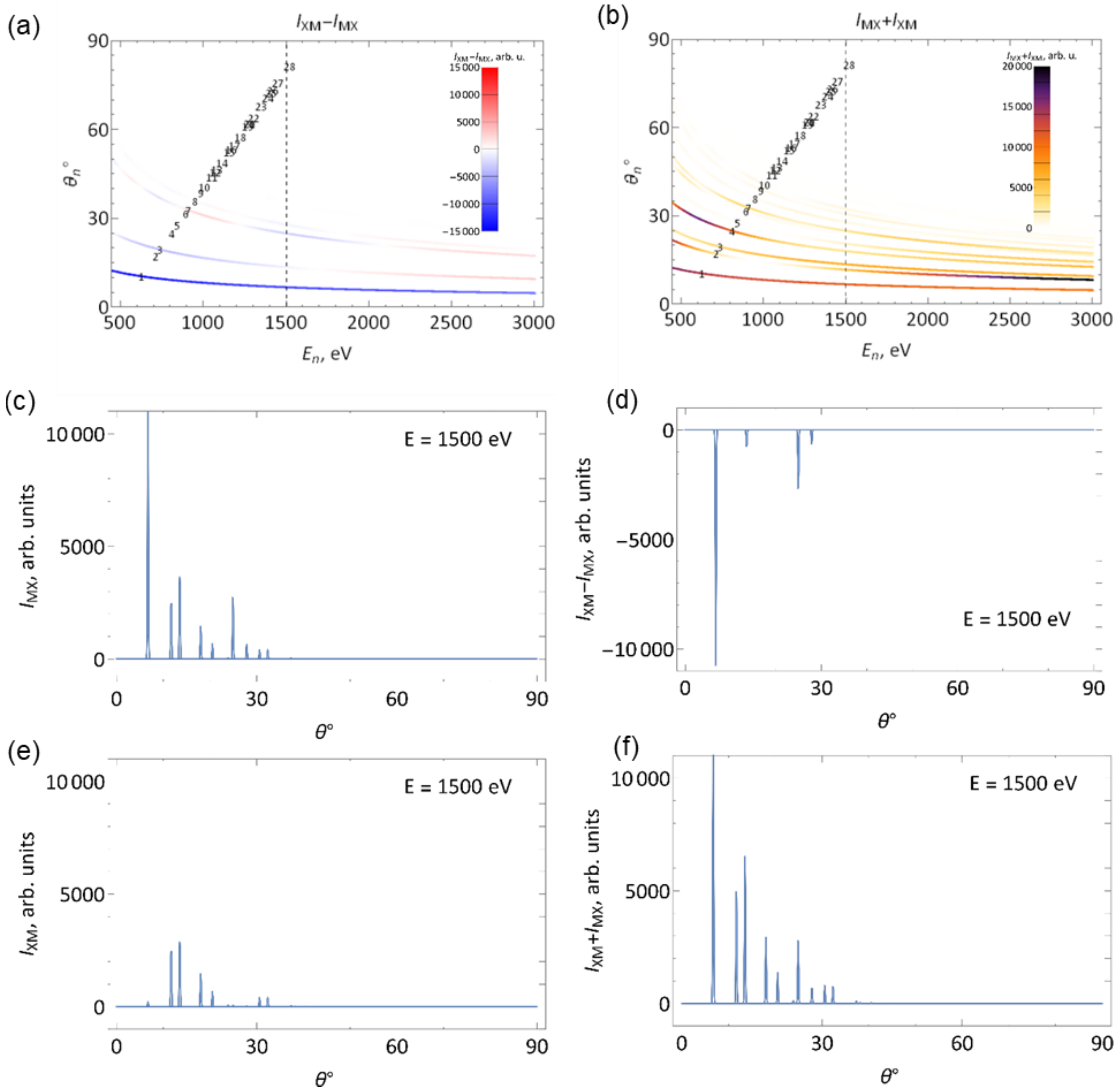

Figure S3.4: a) & **b**), The difference and sum of the transmitted scattering intensity for the two domains, I_{XM} & I_{MX}, both as a function of primary electron beam energy En (eV), respectively. This data was used to calculate the theoretical Michelson contrast for scattering in the range of 0°< θ < 90° (transmission). A colour scale is used to show the scattering intensity where white equates to no intensity. Dashed vertical line highlights the incident electron beam energy of 1500 eV relevant to **c**)-**f**). **c**) & **e**) Intensity of the electron scattering as a function of scattering angle at a primary electron energy of 1500 eV for the MX' and XM' domains, correspondingly. **d**) & **f**) The difference and the sum of the scattering intensity for the two domains, I_{XM}& I_{MX}, both at 1500 eV, respectively.

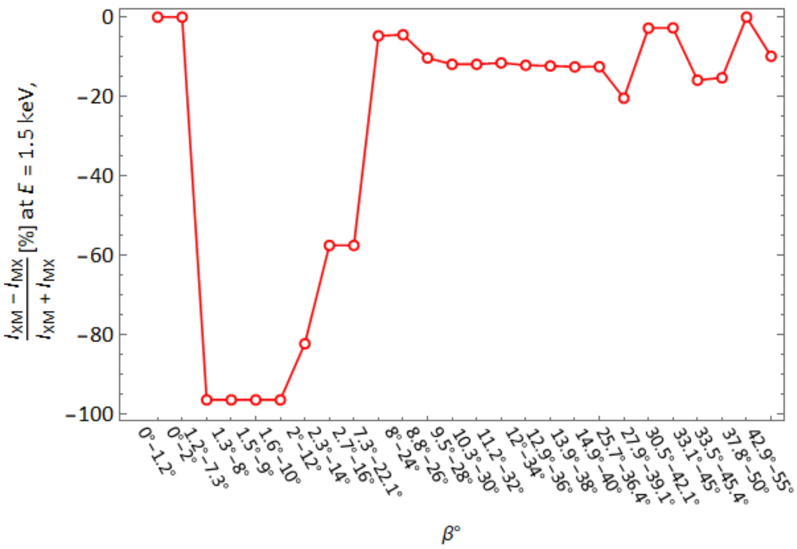

Figure S3.5: Summation of the Michelson contrast for transmitted electrons due to elastic scattering at an incident beam energy of E=1500 eV for different annular intervals of the scattered collection angle.

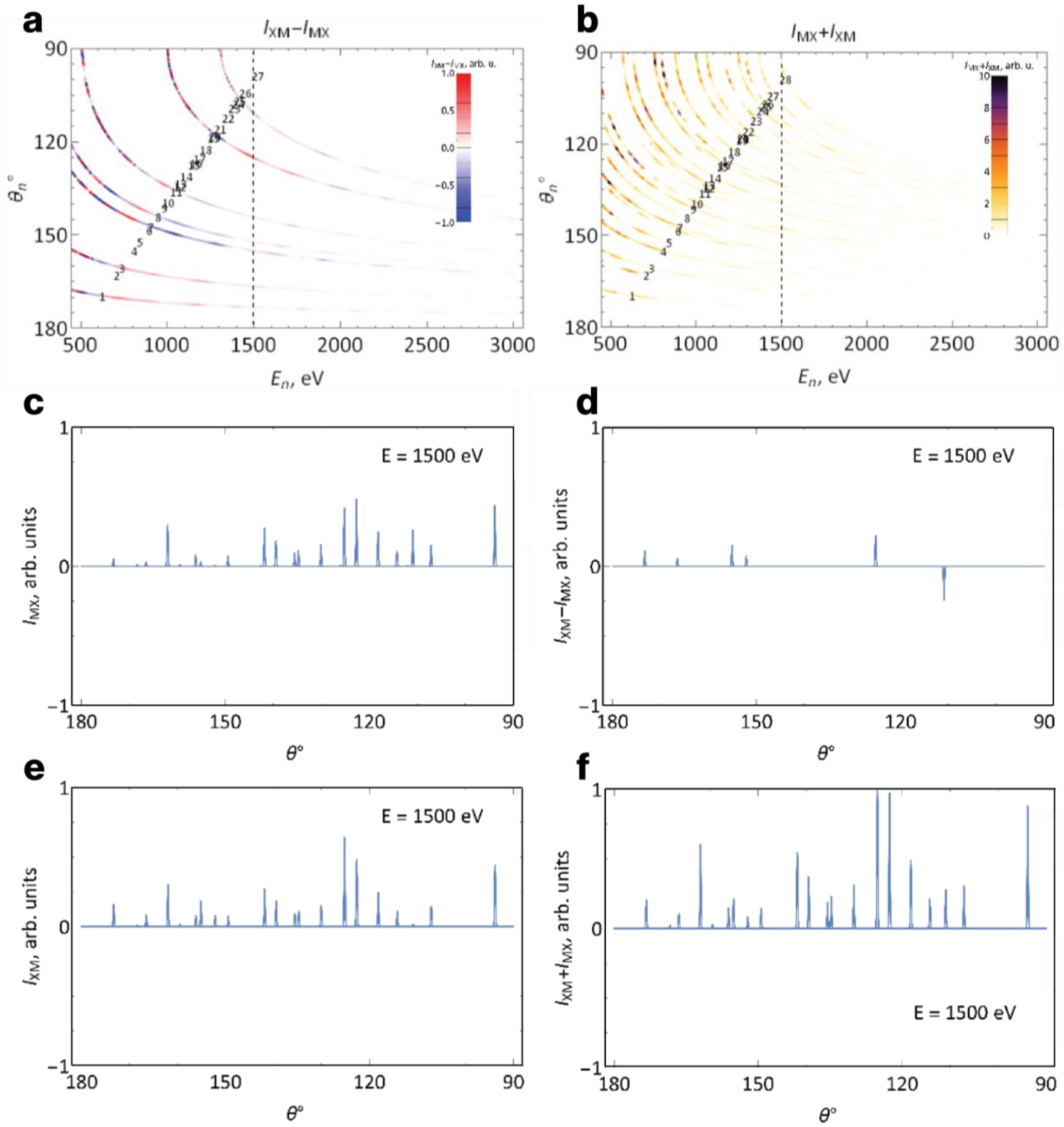

Figure S3.6: **a**) The difference, and **b**) the sum of the reflected scattering intensity for the two domains, I_{XM} & I_{MX}, both as a function of primary electron beam energy E_n (eV) used to calculate the theoretical Michelson contrast for scattering in the range of 90°< θ < 180° (reflection). A colour scale is used to show the scattering intensity where white equates to no intensity. Dashed vertical line highlights the experimental incident electron beam energy of 1500 eV used in this work, at which the intensity plots in **c**)-**f**) were calculated. **c**) & **e**) Intensity of the electron scattering as a function of scattering angle for the MX' and XM' domains, respectively. **d**) & **f**) The difference and the sum of the scattering intensity for the two domains, I_{XM}& I_{MX}, respectively*.* The data is similar to **Figure S3.4** but for reflection elastic processes, the arbitrary units (a.u.) are the same as in **Figure S3.4** demonstrating the magnitude of scattering is ~10^4 times lower.

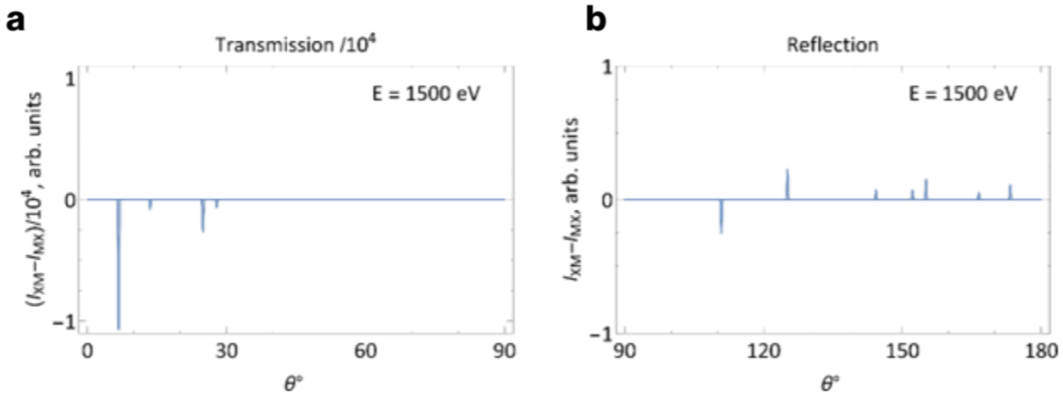

Figure S3.7: Comparison of **a**) transmission, and **b**) reflection domain contrast intensities for Bragg scattering at an incident electron beam energy of E=1500 eV. Note that the x-axis is flipped from **Figure S3.6** so theta increases from left to right. Also worth noting is the factor of 10^4 difference between the transmitted and reflected intensity scales.

3.2. Inelastic Scattering

To understand inelastic scattering of high energy incident electrons, we have constructed a simple Monte Carlo model of secondary electron generation and attenuation. This requires understanding of the underlying processes through which primary electrons transfer energy and momentum to bound TMD valence and core electrons, and the associated cross sections and inelastic mean-free paths (IMFPs). These processes fall into two categories, involving inelastic scattering of the primary beam with *valence* and *core* electrons.

3.2.1. Inelastic loss processes

The valence electron differential IMFP, $\bar{\mu}(E_0, \omega)$, for a primary beam energy, E_0 and energy loss ω, can be calculated from the electron energy loss function, defined as the imaginary part of the inverse dielectric function, $Im\left[-\frac{1}{\epsilon(q,\omega)}\right]$. This quantifies the probability that a primary electron will exchange a given energy and momentum with a materials' valence band, mainly through direct interband transitions and plasmon generation. [3,4] The IMFP is calculated from the energy loss function by integration over all allowed energy and momentum exchanges, $\mu(E_0) = \int_0^E \bar{\mu}(E_0, \omega) \propto \int_{-q}^{q} Im\left[-\frac{1}{\epsilon(q,\omega)}\right]$.

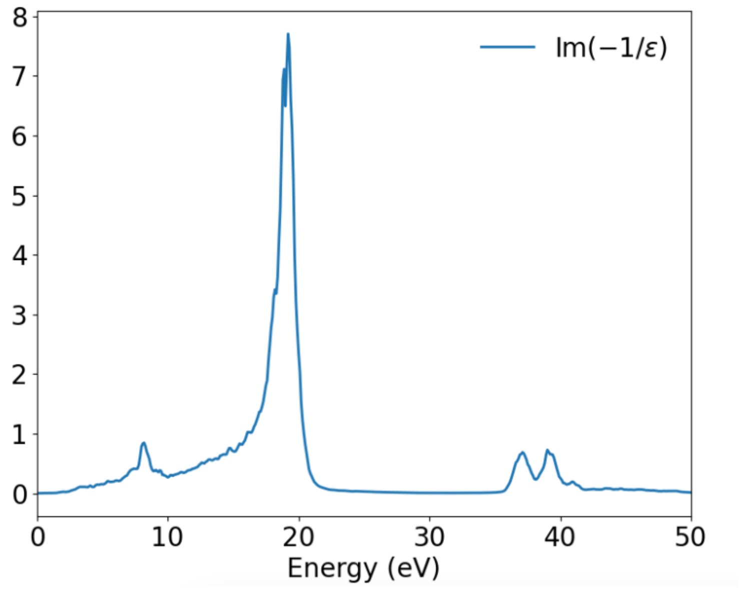

Figure S3.8*:* DFT-calculated energy loss function $Im\left[-\frac{1}{\epsilon(q=0,\omega)}\right]$ for an MoS_2 P-bilayer.

To quantify the energy loss spectrum, $Im\left[-\frac{1}{\epsilon(q,\omega)}\right] = \frac{\epsilon_2}{\epsilon_1^2 + \epsilon_2^2}$ was evaluated using density functional theory (DFT) calculations of the real (ϵ_1) and imaginary (ϵ_2) parts of the dielectric

function, and is shown in **Figure S3.8**. This shows that valence band inelastic scattering is dominated by small energy losses of the primary beam, with maximum energy loss of around 40 eV from a single collision, and peaks at ~8 eV and ~20 eV which correspond to interband transitions and plasmon loss.[3,4]

For semiconducting materials, such as those considered in this work, the core electron contribution to the IMFP is generally less significant than that from valence electrons, contributing to approximately 10 - 20% of the total IMFP for a primary energy ≤ 1500 eV. The differential IMFP for ionisation by an inner shell electron has a sharp edge at the corresponding binding energy, which for the elements considered here fall in the range of 200-500 eV. Core electron ionisation allows for less frequent, larger energy transfer from the primary beam to the bound electrons.[3,4]

3.2.2. Inelastic scattering mean-free paths

Based on these considerations, most electrons generated by scattering by the incident beam will have low energy (< 40 eV), with maximum possible energy transfer of around 500 eV for a single collision (the binding energy of the Mo $M_1$3s orbital, which is the largest sub-1500 eV core electron in MoS_2). The energy dependence of the electron-electron IMFP (λ_{mfp}) in the energy range 50-2000 eV can be calculated using the TPP-2M modification of the Bethe equation[5,6] for energy loss of fast charged particles when passing through matter *via* ionisation.[3,4] This takes the form,

$$\lambda_{mfp} = \frac{E}{E_p^2[\beta \ln \gamma E - C/E + D/E^2]}; \textbf{(8)}.$$

Here, λ_{mfp} is the IMFP in Å, E is electron energy in eV, $E_p = 28.8(N_v\rho/M)$ is the free-electron plasmon energy, ρ is bulk density in gcm^{-3}, N_v is the number of valence electrons. M is molecular weight, E_g is the band gap, and β and γ are additional empirical, material-dependent parameters. Values of λ_{mfp} (in Å) for the electron energy range *E* = 500 to 2000 eV, for MoS_2, WS_2 & hexagonal boron nitride (hBN), using the parameters shown in **Table 1**, are plotted in **Figure S3.9**. The obtained dependence agrees well with available experimental data for the IMFP in MoS_2[7,8] and hBN[9,10] in the 50-2000 eV range.

	**Density, ρ (g/cm³)**	**No. of valence electrons, N_v**	**Band gap energy, E_g (eV)**	**Free-electron plasmon energy, E_p (eV)**
**MoS_2**	5.06	18	1.8	21.72
**WS_2**	7.5	18	2.1	21.25
**HBN**	2.2	8	5.955	24.26

Table 1: TPP-2M parameters for MoS_2, WS_2 and HBN.

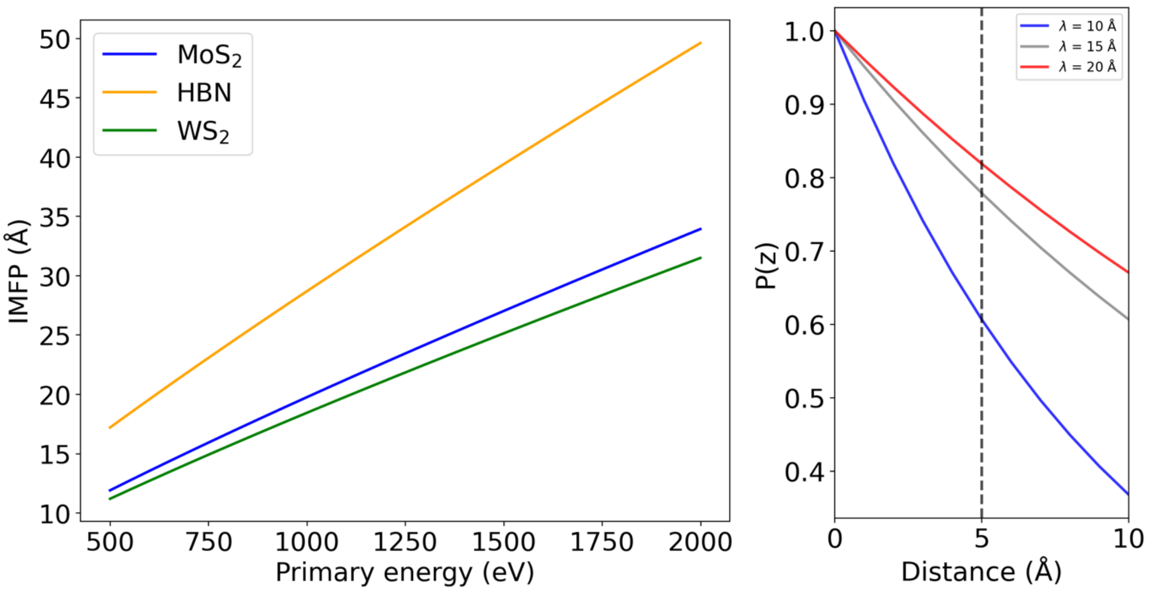

Figure S3.9: λ_{mfp} (IMFP) vs electron energy for MoS_2, WS_2 & HBN (left). Probability an electron travels a distance z, $P(z) = e^{-z/\lambda_{mfp}}$ through an isotropic material with the approximate height of an MoS_2 bilayer for different λ_{mfp} in the 500-2000 eV range of MoS_2 (right).

3.2.3. Monte Carlo modelling

Based on the above considerations, electrons with energies in the range 25 - 500 eV are most relevant to the measured inelastic signal. Emission of an inelastically scattered electron is considered as a two-step process, where an incident primary electron generates secondary electrons from a point $\boldsymbol{r'}$ within the bilayer with a probability proportional to the local electron density $\propto \rho(r')$ (see **Figure S3.1**), which can then be attenuated by the electron density along

the outgoing escape path. The total intensity of secondary electron emission along this path is taken to be a Beer-Lambert style law,

$$I = I_0\, \sigma_{1.5keV} \int d\boldsymbol{r}'\, \rho(\boldsymbol{r}')\ exp\left(-\, \sigma_{low-E} \int ds\, \rho(\boldsymbol{r}' + s[\vec{\boldsymbol{x}}\, \sin\theta cos\phi + \vec{\boldsymbol{y}}\, sin\theta sin\phi + \vec{\boldsymbol{z}}\, cos\theta])\right);\ \textbf{(9)}.$$

where the first integral in this expression is over the full distribution of three-dimensional electron density (which can be sampled by a Monte Carlo algorithm) and *s* is the coordinate along the emission path, which is along a direction with a given tilt (θ) and azimuthal (ϕ) angle.

Low energy attenuation cross sections have been applied to (on average) reproduce the TPP-2M inelastic mean free path for MoS_2[11,12] for a given energy (see **Figure S3.10**). Local electron densities are evaluated using the universal Thomas-Fermi equation, which is solved numerically and used to generate the corresponding electron densities for the atomic species Mo and S, ρ_{Mo} and ρ_S,[13] which are to create a three-dimensional grid of electron density, $\rho(x, y, z)$, at DFT-relaxed lattice positions of the two-dimensional MoS_2 lattice.

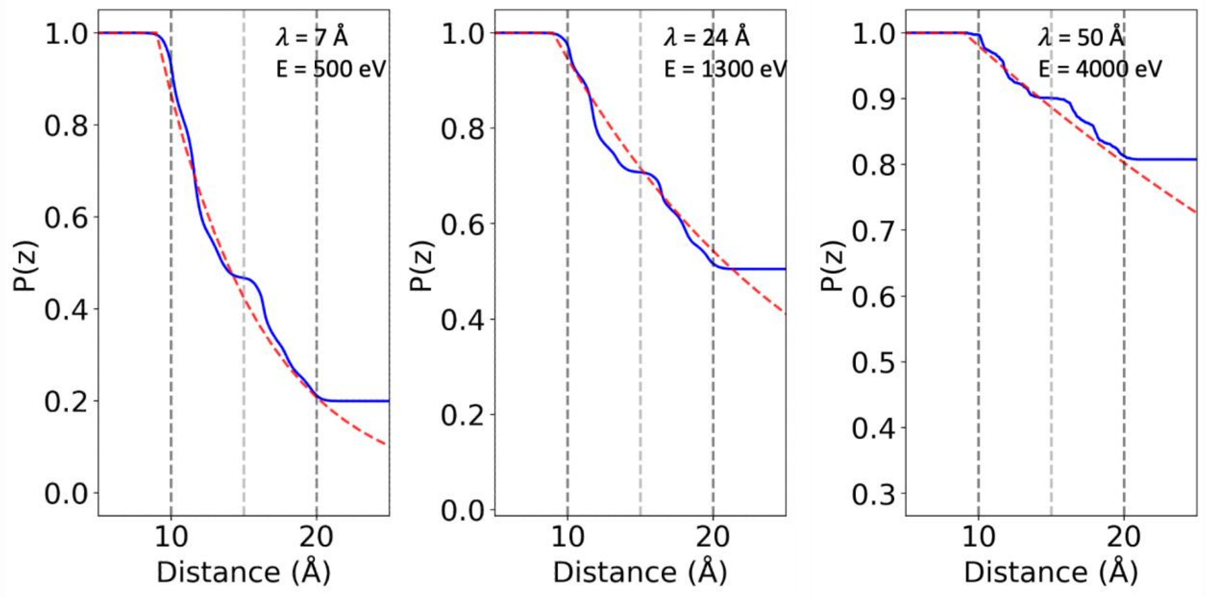

Figure S3.10: Probability a primary electron travels a distance z, through an MoS_2 bilayer, from Monte Carlo simulation of Eq. 9, averaging over 10,000 realisations (blue), and interpolated to the Beer-Lambert exponential dependence, $P(z) = e^{-z/\lambda_{mfp}}$ (red), which enables estimation of λ_{IMFP} as a function of electron energy.

Raw emission data (I_{MX}, I_{XM}), their difference, $I_{XM} - I_{MX}$ and sum, $I_{XM} + I_{MX}$, as a function of emission scattering angle, are shown in **Figure S3.11**. Emission directions which are along the armchair, $\phi = 30^o$, and zigzag $\phi = 60^o$ axes (**Figure S3.12**) are referred to as

“transmitted” and “reflected” for emission angles at θ = 0° and 180° to the assumed primary beam direction, respectively. The average contrast, calculated from summing the intensity from both scattering directions is shown in **Figure S3.13**, along with the reflected signal from inelastic electrons generated in the bilayer (a mirror reflection of the forward scattered signal). **Figure S3.14** presents the resulting STEM detector signal (the electron signal from **Figure S3.13** resolved into the relevant experimentally measured annular ranges).

Figure S3.15 shows the normalised emission intensity as a function of azimuthal angle, ϕ, from an MX domain for fixed scattering angle $\theta\, from\, 150^o\, to\, 160^o$, demonstrating a characteristic twist-dependence of emitted secondary electrons. **Figure S3.16** shows the calculated variation in contrast of the inelastic signal for three different secondary electron energies ($E = 25, 100, 800$ eV) using a fixed primary beam energy of $E_0 = 1500$ eV. We observe significantly greater contrast for low energy secondary electrons.

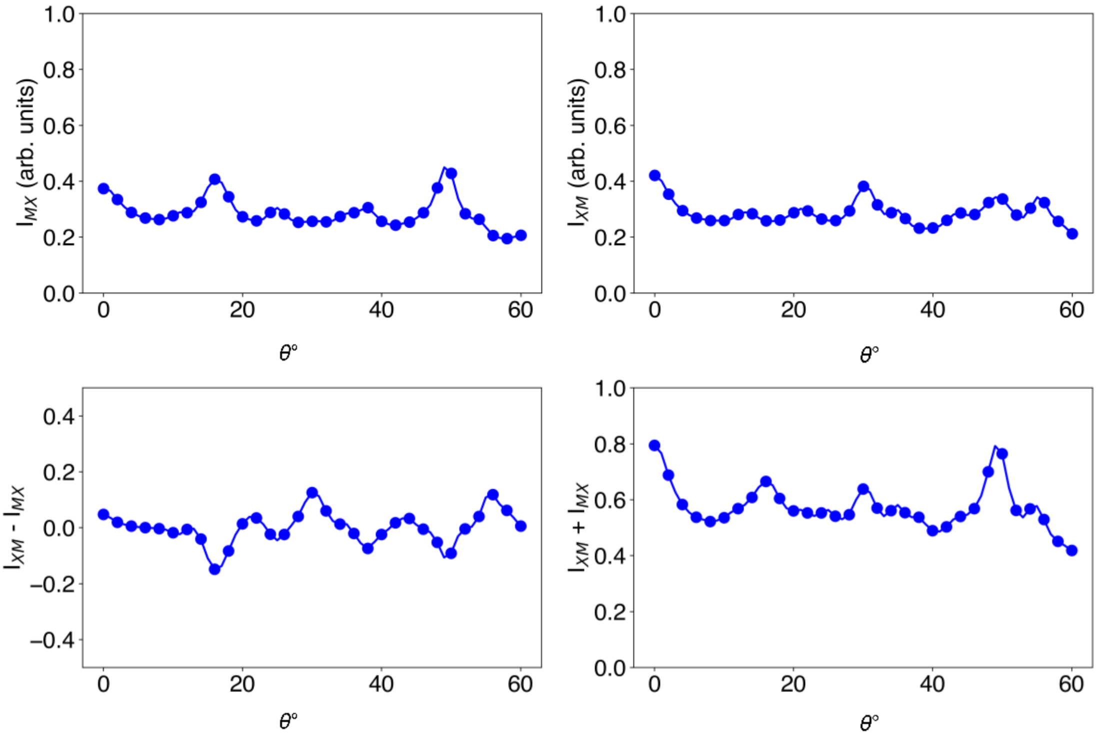

Figure S3.11: I_{XM} intensity and I_{MX} intensity, as well as the domain contrast I_{XM} - I_{MX} and sum intensity I_{XM} + I_{MX}, for inelastic scattering along the **zigzag** direction *($\phi = 60^o$)* using an incident electron beam energy of 1500 eV for secondary electrons with E = 100 eV.

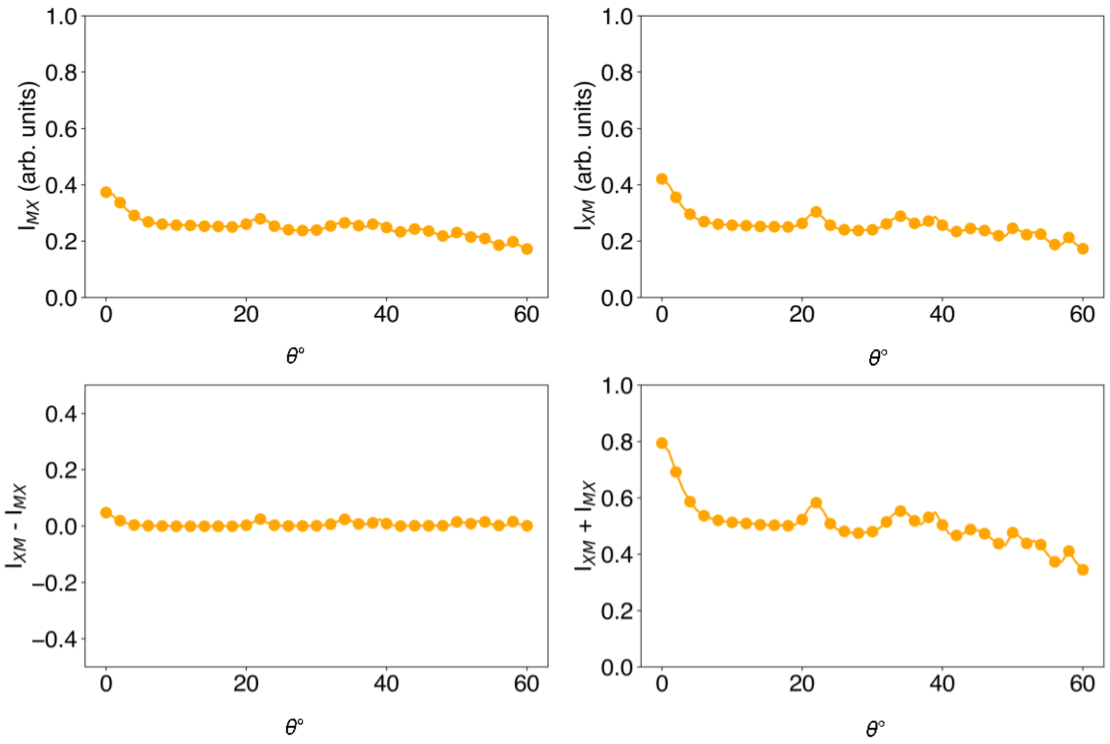

Figure S3.12: I_{XM} intensity and I_{MX} intensity, as well as the domain contrast I_{XM} - I_{MX} and sum intensity I_{XM} + I_{MX}, for inelastic scattering along the **armchair** direction ($\phi = 30^o$) using an incident electron beam energy of 1500 eV and for secondary electrons with E = 100eV.

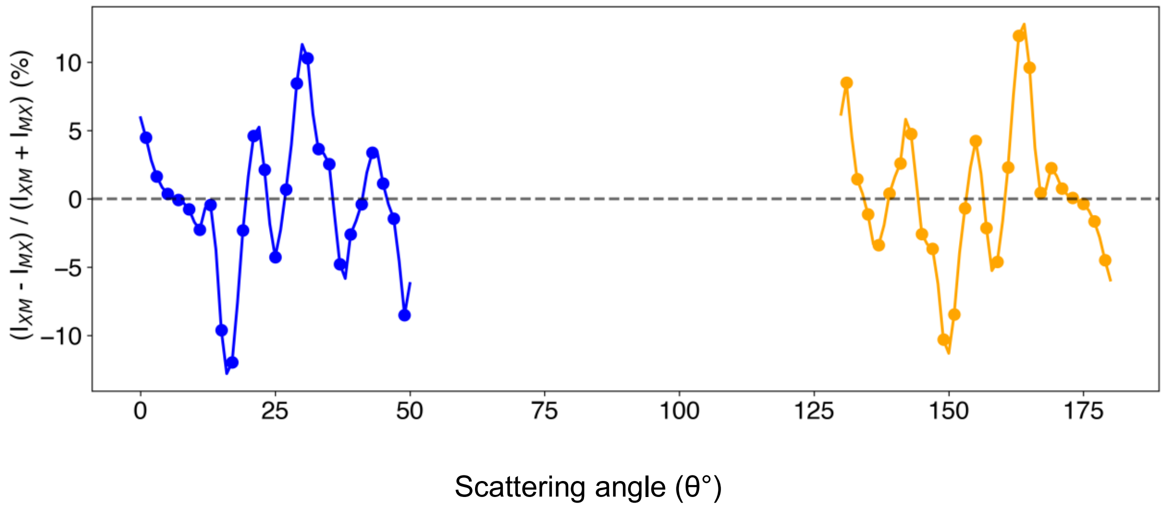

Figure S3.13: Theoretical calculations of Michelson intensity contrast of domains (I_{XM} - I_{MX} / I_{XM} + I_{MX}) vs emission angle for transmitted, inelastically scattered electrons (blue), and reflected inelastically scattered electrons (yellow) averaged over both crystallographic directions for generated electrons with E = 100 eV, and a primary beam energy E_0 of 1500 eV.

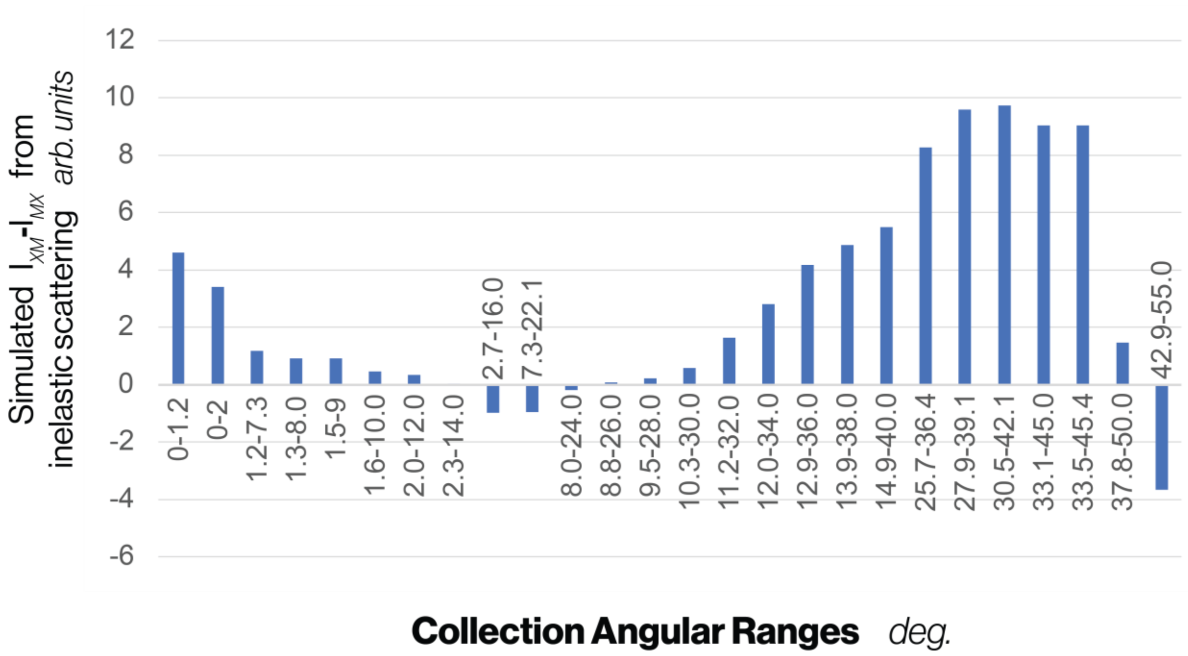

Figure S3.14: I_{XM} - I_{MX} (arb units) from the inelastic scattering processes shown as a function of scattering angle for the experimental annular ranges of collection angle (annular range in degrees) for the different STEM detector configurations (similar to **Figure S3.5** but for inelastic scattering).

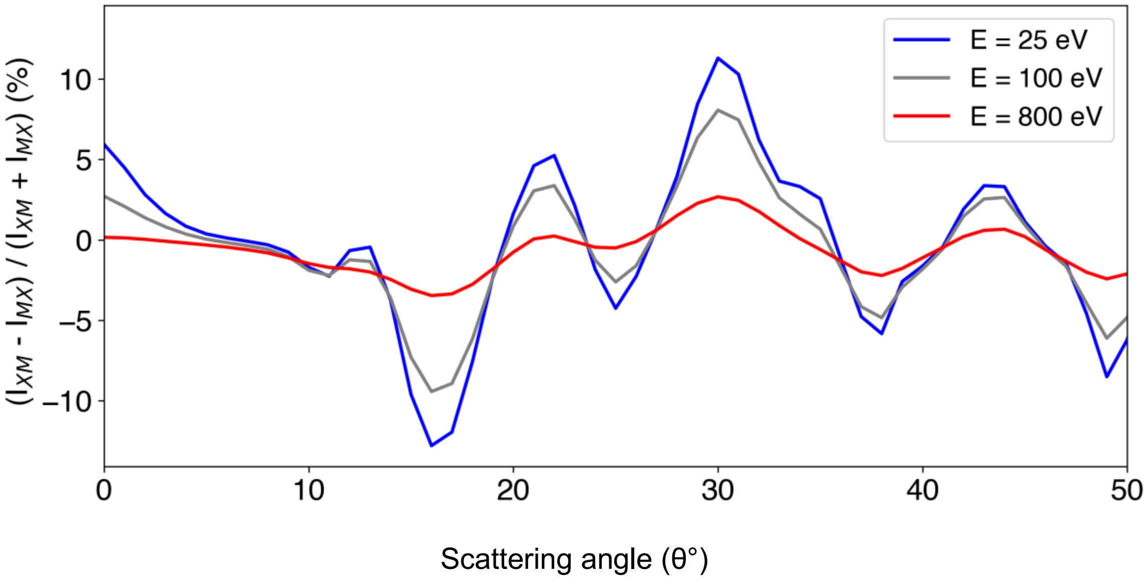

Figure S3.15: Azimuthally averaged (ϕ-averaged) theoretical calculations of Michelson intensity domain contrast (I_{XM} - I_{MX} / I_{XM} + I_{MX}), calculated for a primary beam energy E_0 = 1500 eV and secondary electron energies E = 25, 100 and 800 eV. The 100-eV data is the same as that shown in **Figure S3.13**.

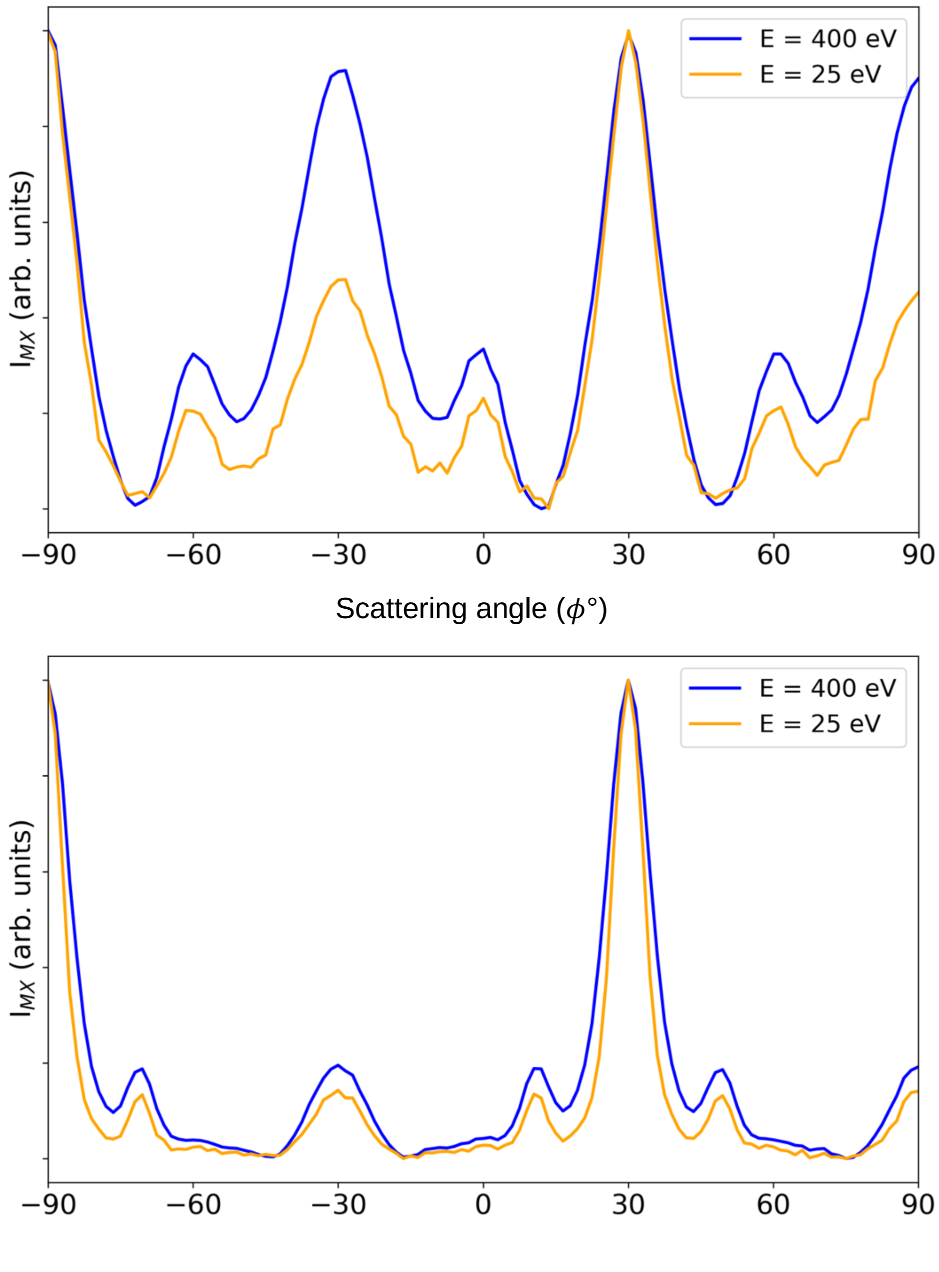

Scattering angle (ϕ°)

Figure S3.16: Emission intensity vs in plane azimuthal angle, ϕ, for a fixed scattering angle from the transmitted beam of $\theta = 160^o$ (top) and $\theta = 150^o$ (bottom).

3.2.4 Angular dependence

Inelastic scattering can also be well-approximated by the double differential cross-section, for an electron of energy E_0 to lose energy ω, deflected by an angle θ,

$$\frac{\partial^2\sigma}{\partial\omega\partial\Omega} \propto \frac{1}{E_0}\frac{1}{\theta^2+\theta_E^2}\frac{\omega E_P^2\Delta}{\left[\omega^2-E_P^2-4\gamma E_P E_0\left(\theta^2+\theta_E^2\right)\right]^2+\omega^2\Delta^2}; \text{ (10)}.$$

where Δ and γ are plasmon damping and dispersion coefficients and the critical angle is related to energy loss as $\theta_E = \omega/E_0$. θ_E is very small for direct energy loss of the primary beam by valence processes due to the small energy transfers involved.[11,12] **Figure S3.17** shows the angular cross section of a high energy (1500 eV) and low energy (30 eV) electrons scattered by the plasmon peak in MoS_2, which shows a very small angular range of generated electrons directly excited by the primary beam.

High-angle scattering will therefore largely arise from second-generation inelastic scattering of secondary electrons, and from high-energy core scattering processes where primary electrons lose a large proportion of their initial kinetic energy, which have a qualitatively similar cross section to the one depicted in **Figure S3.17**. These processes involve multi-generation scattering and core cross-sections which are not considered by the MC model employed here, which we expect becomes less accurate at high energy and large angles.

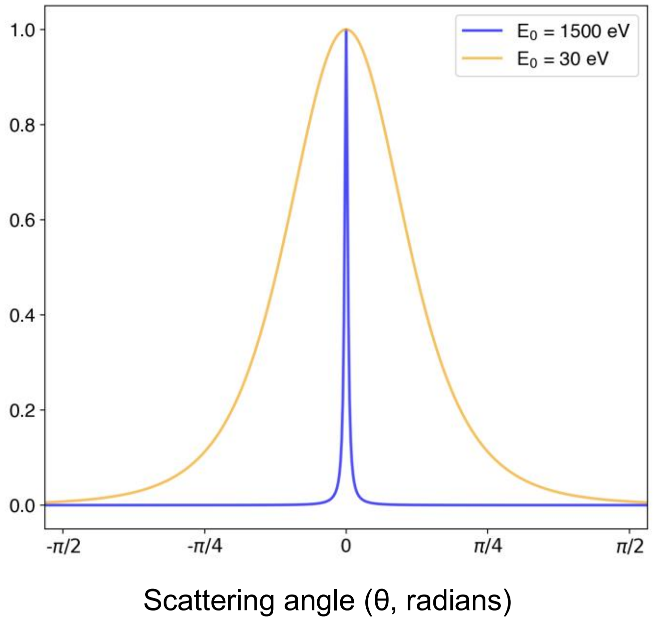

Figure S3.17: Angular dependence of the double differential cross section, integrated over all allowed energies for plasmon scattering of the primary electron beam.

4. Optimising SEM Instrument Parameters

4.1 Domain contrast comparisons with varying SEM parameters

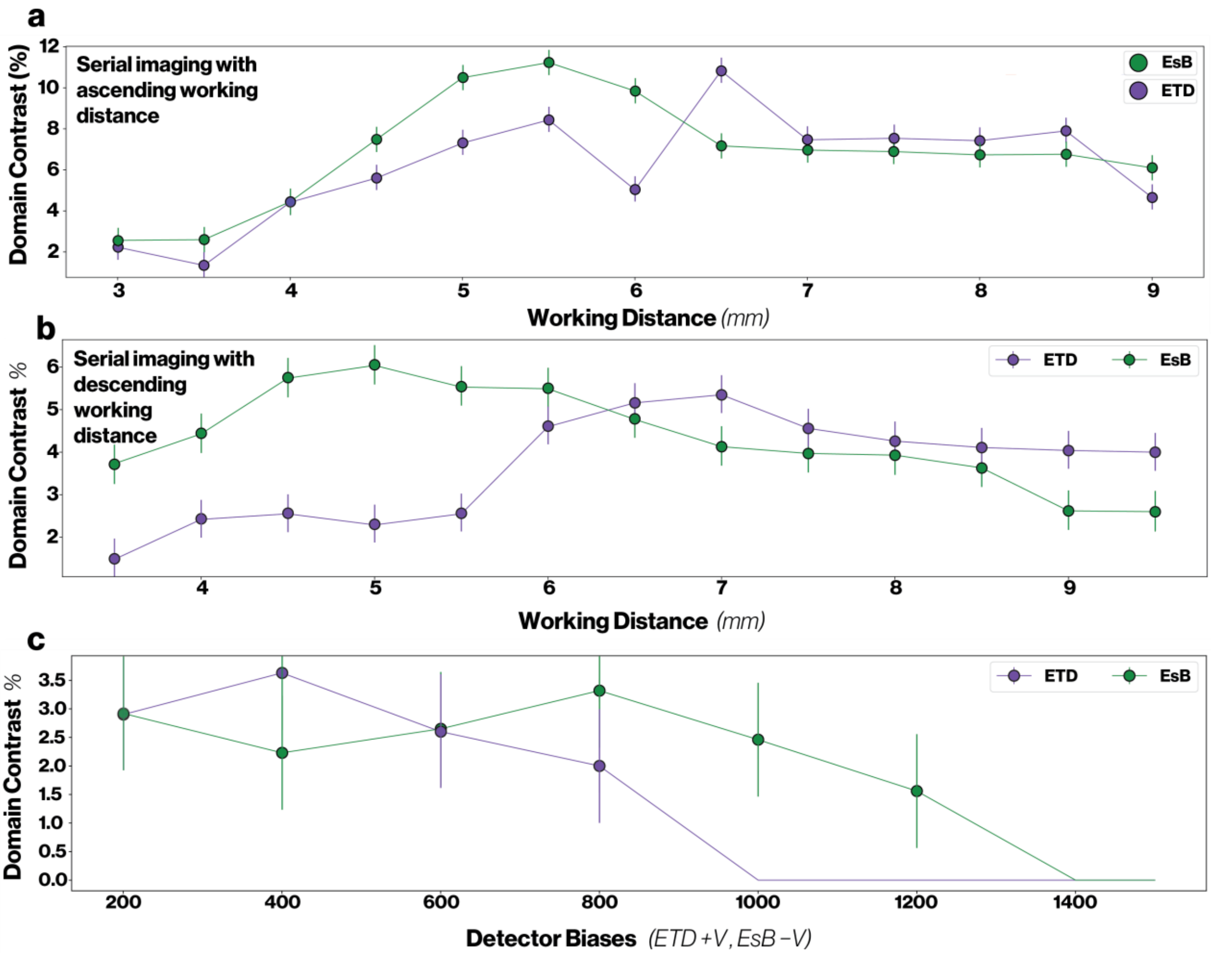

Figure S4.1: Effect of **a**) ascending, and **b**) descending working distance on domain contrast using fixed detector biases of -800 V for EsB and +400 V for ETD. **c**) Effect of detector bias on the domain contrast observed from the ETD and EsB signals when imaging a twisted 3R type MoS_2 bilayer with a fixed working distance of 4.5 mm. An accelerating voltage of 1.5 kV was used for all data acquisition. Note that domain contrast values of less than 4% will be more prone to measurement errors in images with a low SNR and are also more sensitive to the presence of surface contamination, ripples or defects.

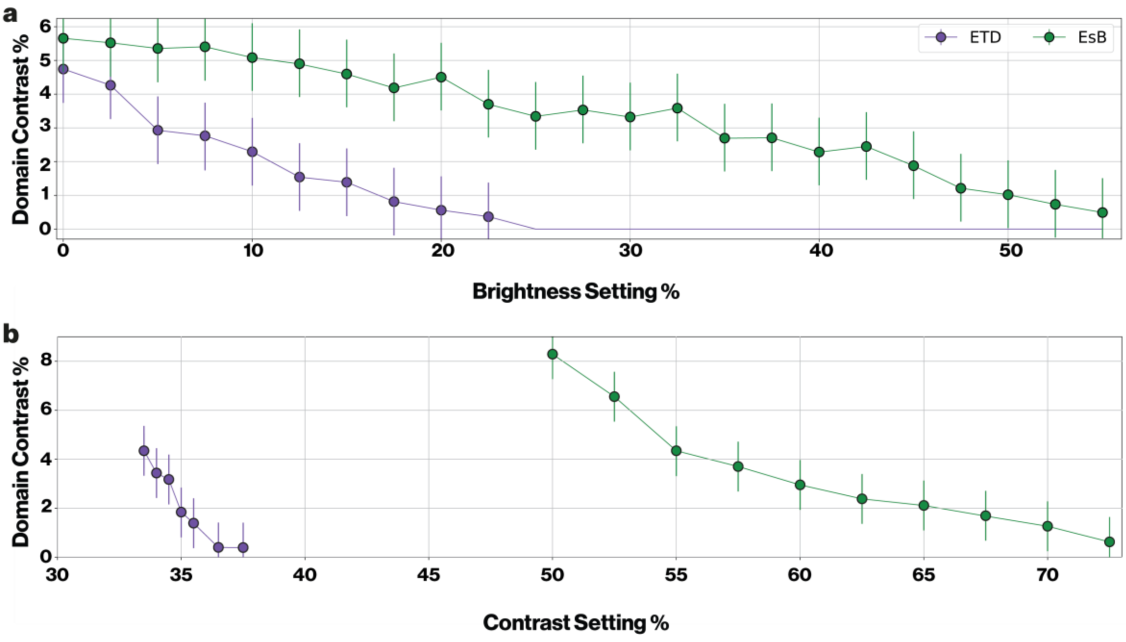

Figure S4.2: Domain contrast measured with varying brightness and contrast settings of the ETD and EsB detectors. The EsB domain contrast is found to be more robust to the precise a) brightness, and b) contrast settings, making domains imaged using this detector more readily observed. All Images used for contrast measurement were acquired at a 25 kX magnification, 5 mm working distance, 1.5 kV acceleration voltage, -800 V EsB detector bias and +400V ETD detector bias. The system vacuum was measured to be 1.4 x 10^{-6} mbar.

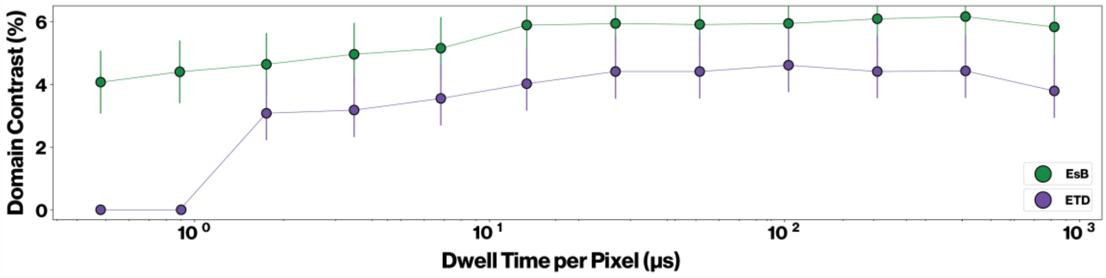

Figure S4.3: Effect of pixel dwell time on the domain contrast acquired by EsB and ETD detectors. EsB imaging has a lower minimum dwell time compared to ETD. Images were all acquired at 25 kX magnification, 5.2 mm working distance, 1.5 kV acceleration voltage, -500 V EsB detector bias and +500 V ETD detector bias. Brightness and contrast settings were 0.6% and 31.4% for the ETD, and 18.1% and 54.3% for the EsB, respectively. The system vacuum was measured to be 1.4 x 10^{-6} mbar.

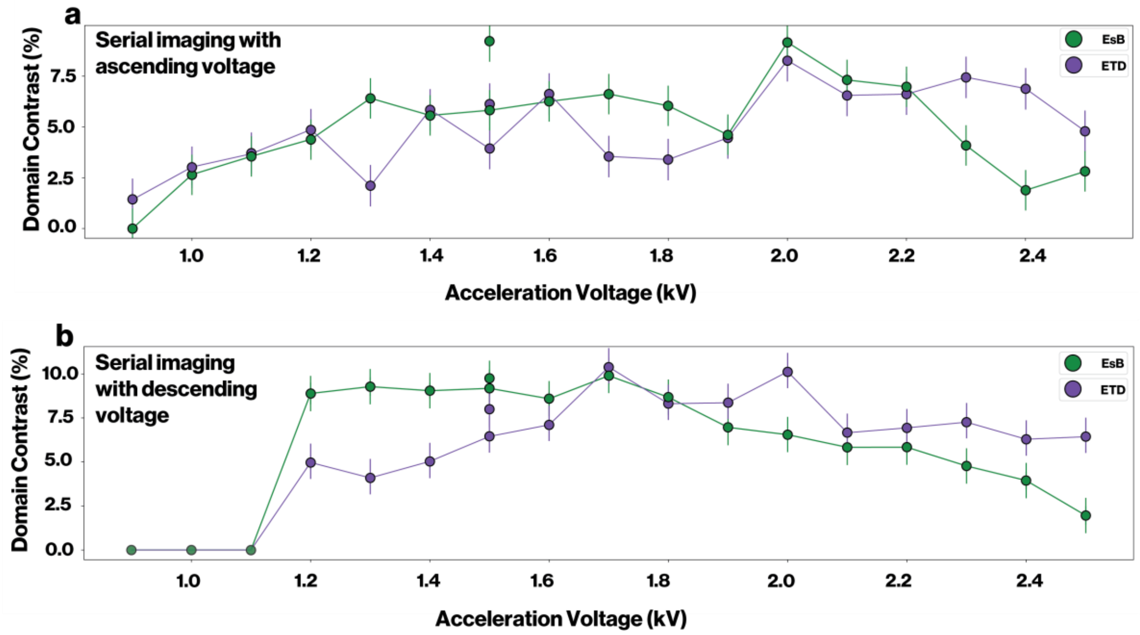

Figure S4.4: Effect of accelerating voltage on domain contrast acquired by EsB and ETD detectors. Serial imaging conducted with a) ascending, and b) descending voltages. The separate data points at 1.5 kV were acquired before starting the image series. The lower contrast values measured from the series data at the same accelerating voltage is due to the build-up of surface contamination.

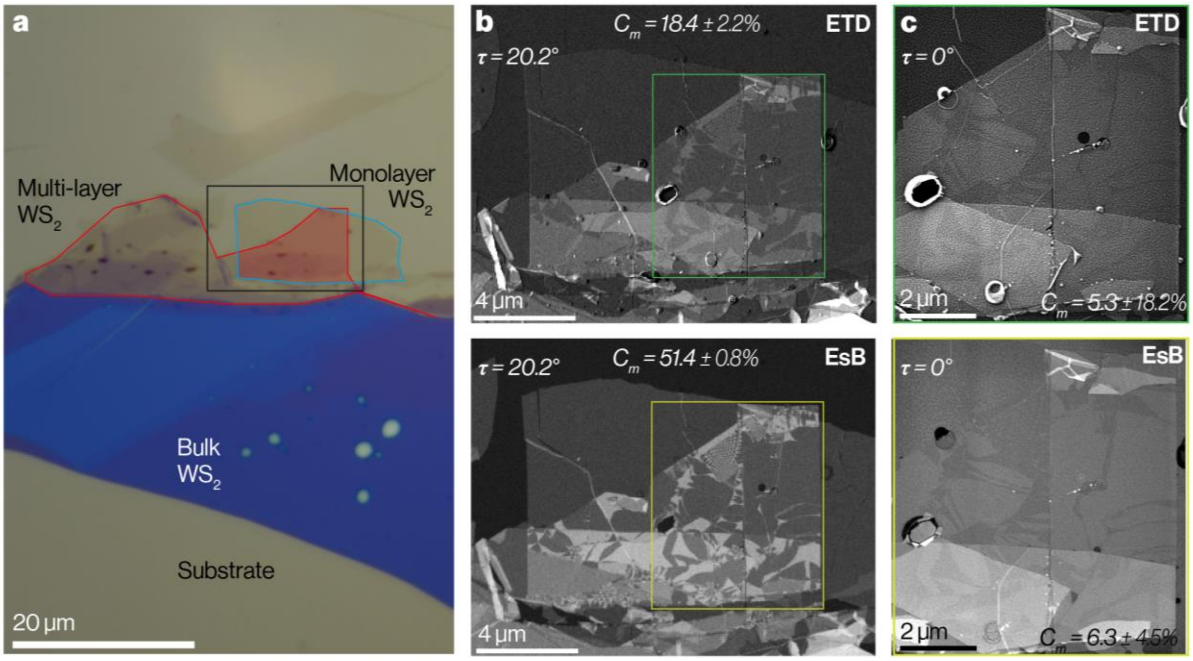

Figure S4.5: Observed domain contrast change with stage tilt for WS_2. a) Optical micrograph of monolayer WS_2 (outlined in blue) placed atop a 1-3 layers thick WS_2 flake (outlined in red). **b,c**) SEM images at b) 20.2° and c) 0° stage tilt for ETD (top) and EsB (bottom) signals. The areas shown in c) correspond to the regions highlighted in b). A contrast inversion can be observed due to stage tilt.

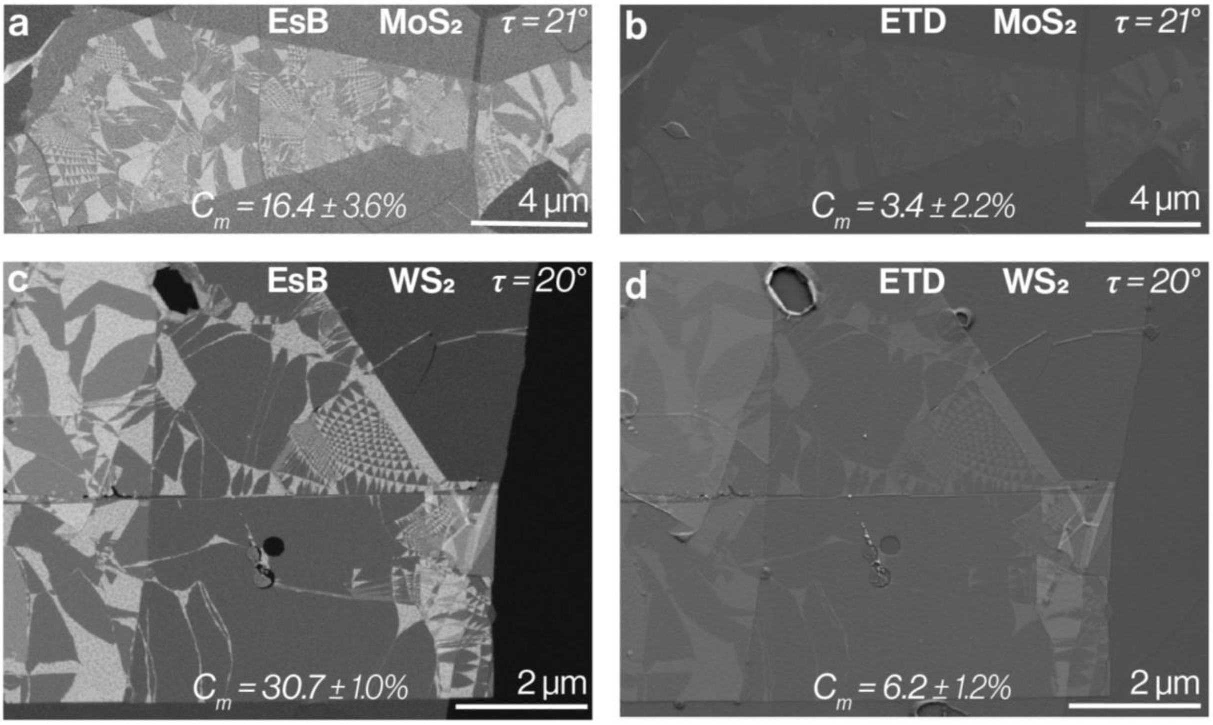

Figure S4.6: Domain contrast comparison between different TMD bilayers. a,b) MoS_2. imaged with the a) EsB, and b) ETD detectors. **c,d)** WS_2. imaged with the c) EsB, and d) ETD detectors. All images were acquired with similar instrumental parameters (WD = 4.2-5.5 mm, acceleration voltage = 1.5 kV, detector biases have been set to -/+ 500 eV for the EsB and ETD electron detectors, respectively).

As the BSE (EsB) detectors are rotationally symmetric, rotation of the sample about the imaging direction, φ, at zero specimen tilt, τ, results in no contrast change (**Figure S4.7**). There is also little change observed from the ETD signal, which is expected as previous work required large specimen tilt to measure azimuthal contrast variations.[14,15]

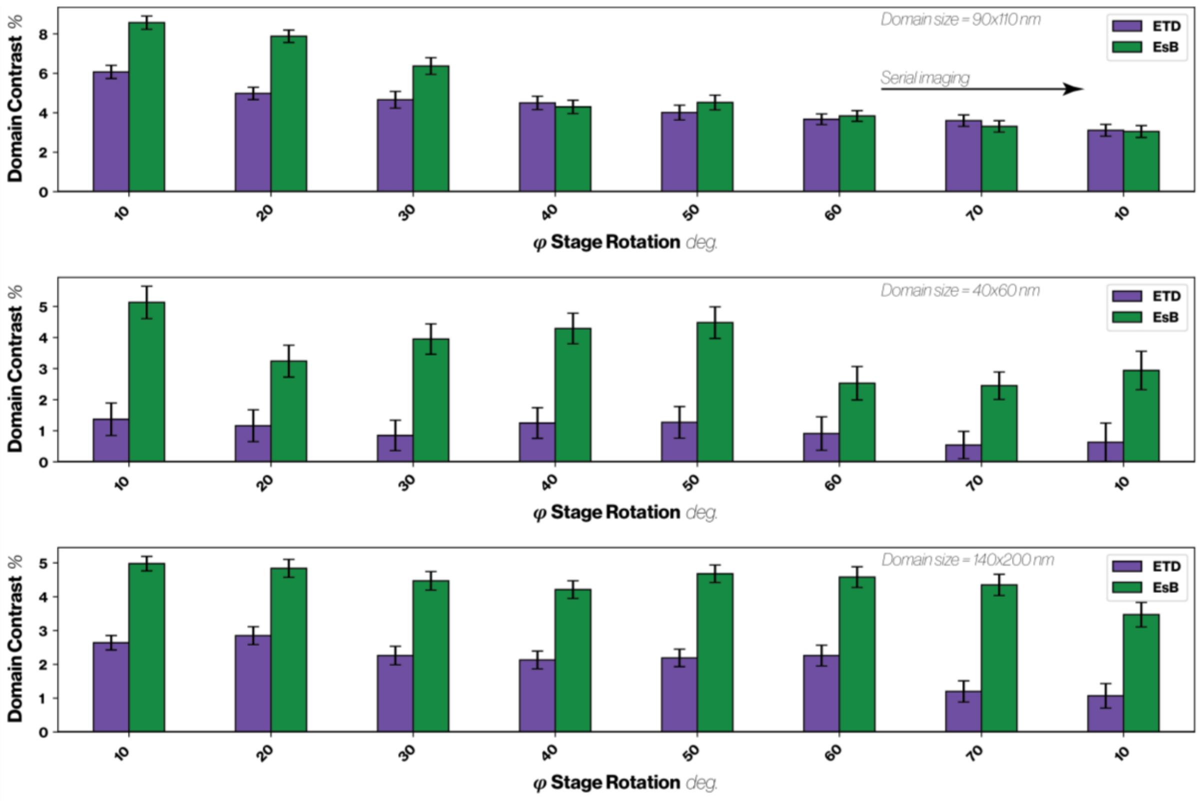

Figure S4.7: Domain contrast change with stage rotation in increments of 10° at 0° tilt for three different samples and different imaging areas (given inset). Note that after 7 images at increments of 10° the right-hand side image is of the sample back at 10°. All data sets showed reduced contrast after the image series which is attributed to the build-up of surface contamination, although the extent of contrast reduction during data acquisition depended greatly on the cleanliness of the sample and SEM chamber. Electron images were acquired at 54 kX magnification, 5.3 mm working distance, 1.5 kV acceleration voltage and a beam current of 1 nA.

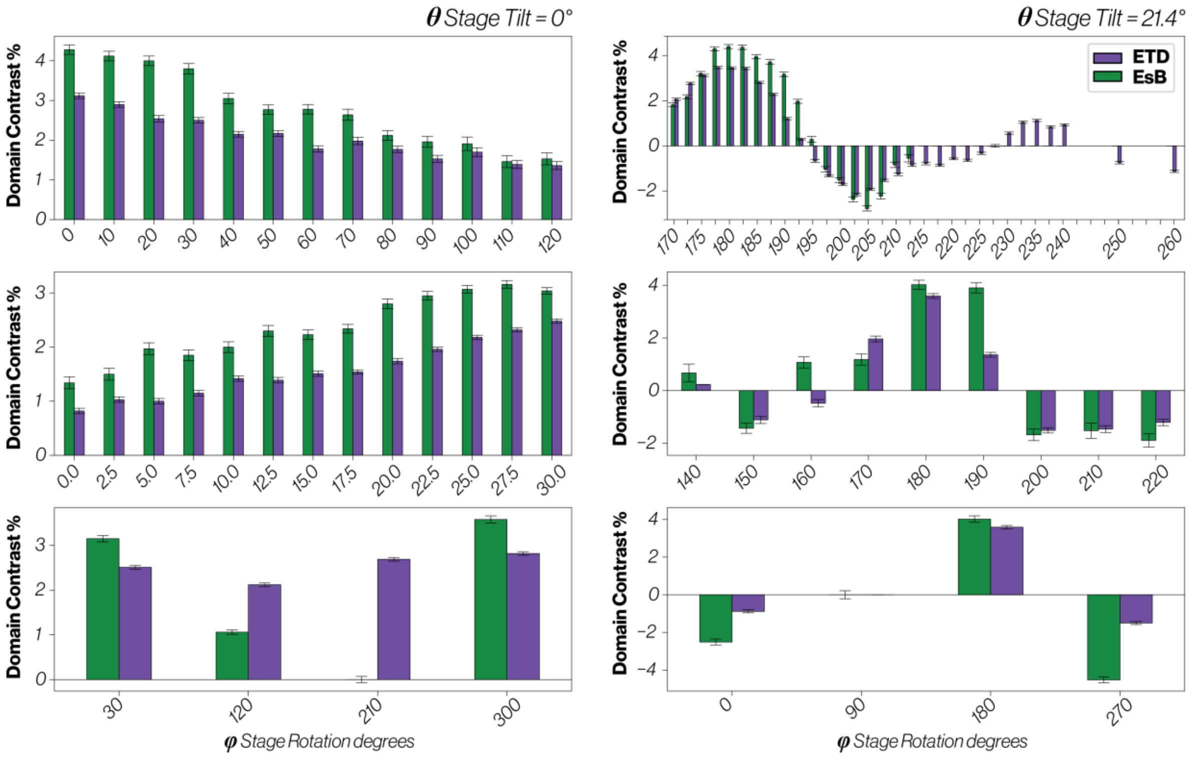

Figure S4.8: Domain contrast change with stage rotation at 0° (left) and 21.4° (right) stage tilt for three different regions of interest within an atomically reconstructed twisted MoS_2 sample. Note that after multiple images all data sets showed reduced contrast which is attributed to the build-up of surface contamination. Electron images were acquired at 54 kX magnification, 5.3 mm working distance, 1.5 kV acceleration voltage and a beam current of 1 nA.

4.2 Effect of beam induced carbon contamination on domain contrast

If the SEM chamber is not ultra-clean there is a tendency for hydrocarbon contamination to build up on the surface, which acts to degrade image contrast. This hinders sequential measurements of domain contrast as a function of tilt angle (as seen in the top row of **Figure S4.7**, where after 7 images the contrast at 10° is significantly poorer than in the first image). The contrast reduction is similar to that seen for the presence of hBN encapsulation (main text, **Figure 5**) where the EsB and ETD domain contrast values show similar amounts of degradation (proportional to their original intensity). Carbon contamination of the surface post imaging was measured using AFM and found to have a thickness of ~1nm after acquiring the data in **Figure S4.7**.

Interestingly, we find that plasma cleaning the SEM chamber with the sample in-situ provides a means of removing both adventitious surface contamination and surface contamination deposited by previous SEM imaging. Plasma cleaning was done at 20 W with N_2 at ~0.3 mbar pressure for 30 minutes using an *in situ* RF Evactron plasma cleaner. We find that this plasma

treatment does not remove the contamination directly but facilitates cleaning of the sample with subsequent SEM imaging as shown in the **Supplementary Video S2** and quantified in **Figure S4.9**. SEM imaging after plasma cleaning was observed to restore the original domain contrast in previously imaged areas where contrast has degraded. After 200 images a ~150% improvement in domain contrast was observed for the previously unimaged areas and ~350% improvement was seen in areas that had been contaminated by previous SEM imaging.

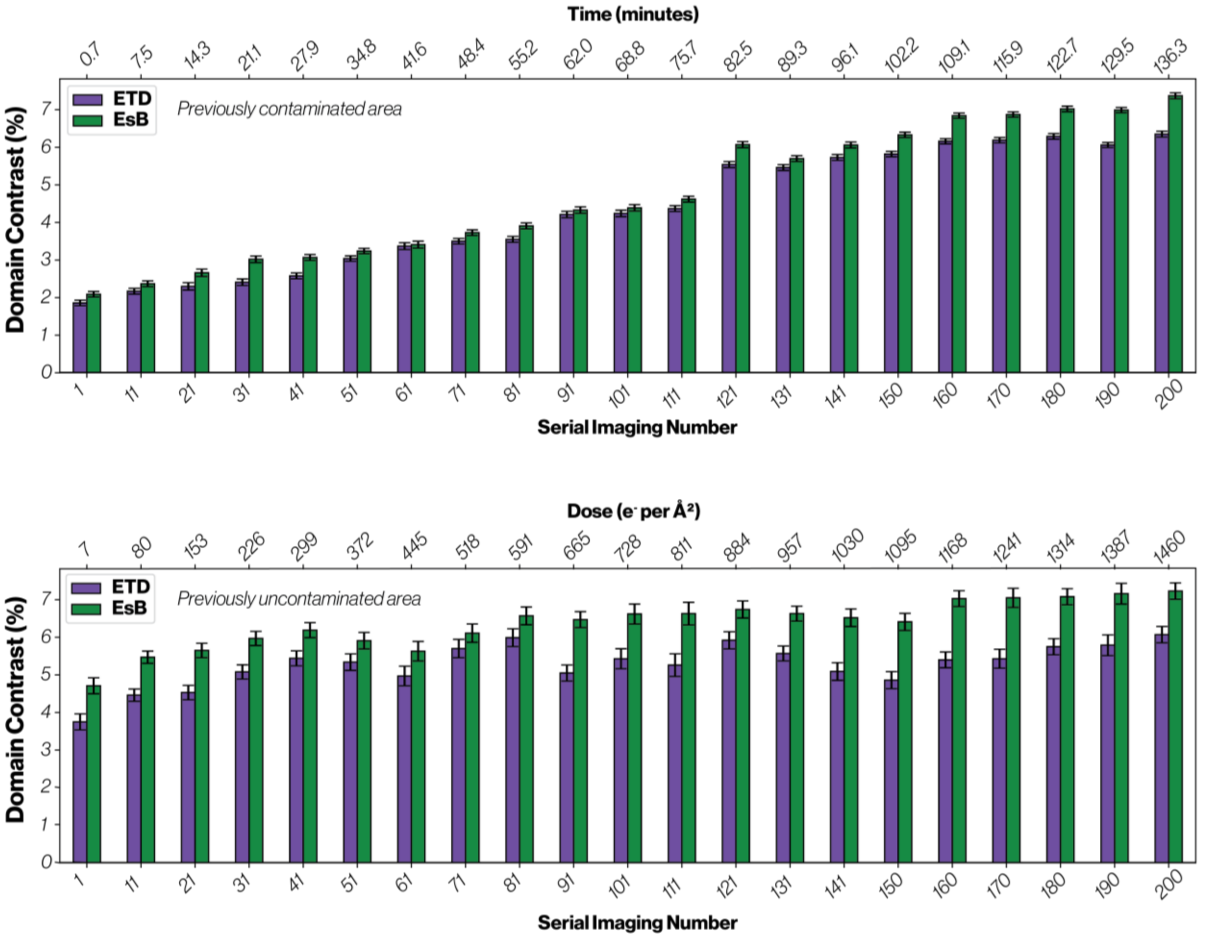

Figure S4.9: Domain contrast as a function of serial imaging after *in situ* plasma cleaning. Domain contrast improves with sequential imaging after *in situ* plasma cleaning of the sample as the electron beam removes deposited surface contamination. Video series is shown in Supplementary Movie S3. Top row shows domain contrast for a previously imaged region and bottom row shows domain contrast for a neighbouring region which has not been previously imaged. A dwell time of 52 µs per pixel was used with a probe current of 1 nA and 1.5 kV acceleration voltage.

4.3 Imaging MoS_2 domain contrast in different SEMs

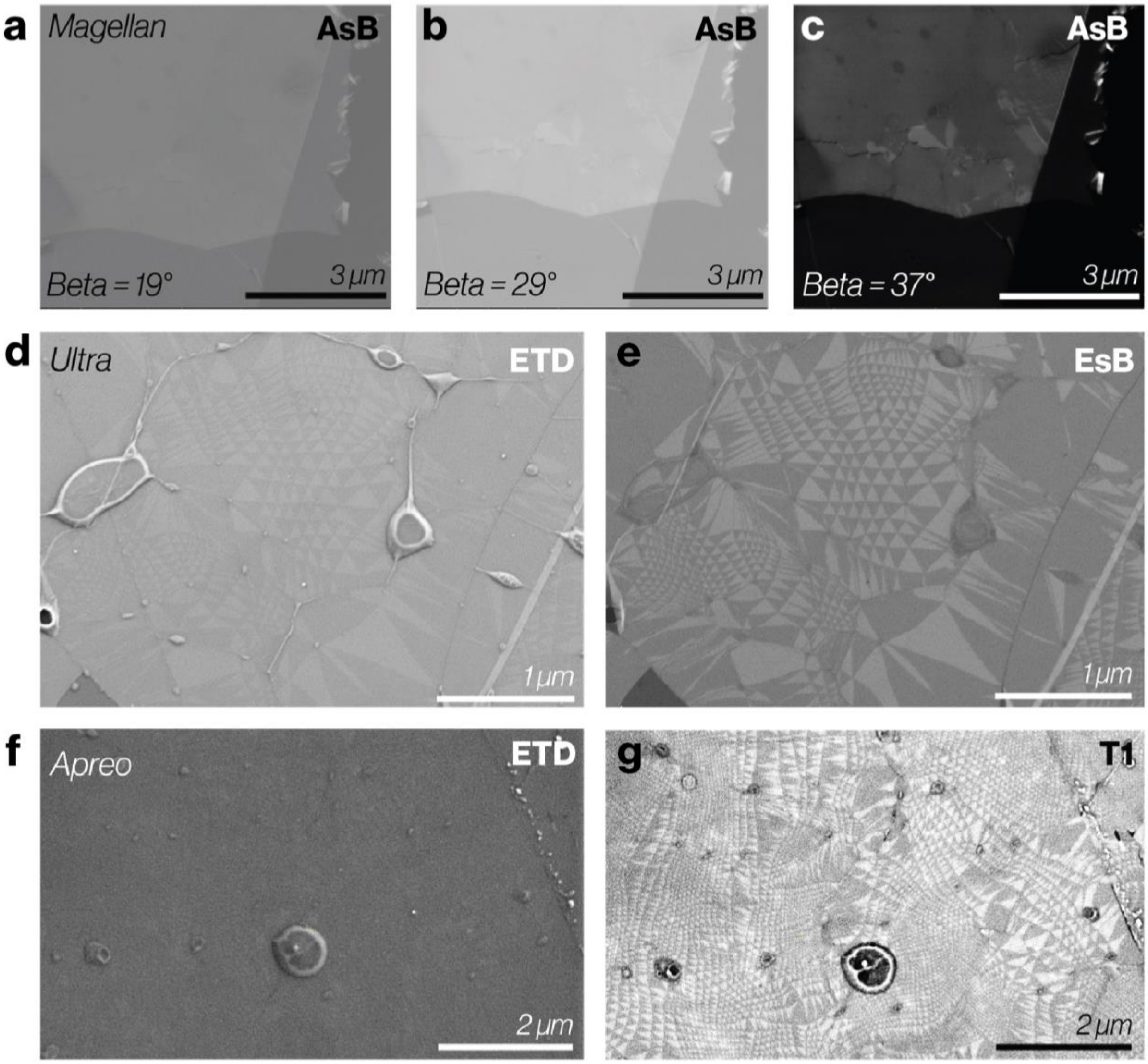

Figure S4.10: Imaging of twisted domain contrast for MoS_2 bilayers using three different SEM columns (all other data presented was obtained on the Zeiss Merlin SEM platform). a-c: FEI Magellan SEM imaging acquired with the angular selective backscatter (AsB) detector at collection inner angles (beta) of 19°, 29° and 37°, (left to right). **d**, **e**: Zeiss Ultra SEM imaging comparing ETD and EsB images. **f**, **g**: FEI Apreo SEM imaging ETD and backscattered electron (T1 detector) images.

Supplementary Video S1

Top ETD and **bottom** EsB electron image tilt series which has been aligned to minimise tilt distortion and drift to highlight changes in domain contrast. Images were acquired at 4.5-8 kX magnification, with a 5.9-6.4 mm working distance and an acceleration voltage of 1.5 kV.

Supplementary Video S2

Top ETD and bottom EsB electron image time series showing contrast improvement during SEM imaging. A poorer contrast region on the left-hand side of the image has resulted from SEM imaging in the presence of carbon contamination. The cleaning (rather than deposition) during imaging results from the use of *in situ* plasma cleaning of the sample so that the electron beam sputters away contamination rather than fixing them to the surface.

References

1. Kim, K. *et al.* Van der Waals Heterostructures with High Accuracy Rotational Alignment. *Nano Lett.* **16**, 1989–1995 (2016).

2. Schneider, C. A., Rasband, W. S. & Eliceiri, K. W. NIH Image to ImageJ: 25 years of image analysis. *Nat. Methods* **9**, 671–675 (2012).

3. Tanuma, S., Powell, C. J. & Penn, D. R. Calculations of electron inelastic mean free paths for carbides. *Proceedings, Annu. Meet. Electron Microsc. Soc. Am.* **54**, 130–131 (1996).

4. Shinotsuka, H., Tanuma, S., Powell, C. J. & Penn, D. R. Calculations of electron inelastic mean free paths. XII. Data for 42 inorganic compounds over the 50 eV to 200 keV range with the full Penn algorithm. *Surf. Interface Anal.* **51**, 427–457 (2019).

5. Shimizu, R. & Ze-Jun, D. Monte Carlo modelling of electron-solid interactions. *Reports Prog. Phys.* **55**, 487–531 (1992).

6. Tanuma, S., Powell, C. J. & Penn, D. R. Inelastic Mean Free Paths of Low-Energy Electrons in Solids. *Acta Phys. Pol. A* **81**, 169–186 (1992).

7. Krawczyk, M., Pisarek, M., Szoszkiewicz, R. & Jablonski, A. Surface characterization of MoS2 atomic layers mechanically exfoliated on a si substrate. *Materials (Basel).* **13**, 1–12 (2020).

8. Neu, P. S., Šiškins, M., Krasovskii, E. E., Tromp, R. M. & Van Der Molen, S. J. Electron transmission and mean free path in molybdenum disulfide at electron-volt energies. *Phys. Rev. B* **107**, 75427 (2023).

9. Chang, H. Y., Alvarado, A., Weber, T. & Marian, J. Monte Carlo modeling of low-energy electron-induced secondary electron emission yields in micro-architected boron nitride surfaces. *Nucl. Instruments Methods Phys. Res. Sect. B Beam Interact. with Mater. Atoms* **454**, 14–22 (2019).

10. Sutter, P. & Sutter, E. Thickness determination of few-layer hexagonal boron nitride films by scanning electron microscopy and Auger electron spectroscopy. *APL Mater.* **2**, (2014).

11. Luo, S. & Joy, D. C. Monte Carlo Calculations of Electron Emission at Surface Edges. *Scanning Microsc.* **1**, 951–962 (1987).

12. Liu, T., Temprano, I., King, D. A., Driver, S. M. & Jenkins, S. J. Epitaxial growth of few-layer MoS2(0001) on FeS2{100}. *Chem. Commun.* **51**, 537–540 (2015).

13. Landau, L. D. & Lifshitz, E. M. *Quantum mechanics - Non-relativistic Theory*. *Quantum mechanics - Non-relativistic Theory* vol. 3 (Pergamon Press, ISBN: 0750635398, 1981).

14. Andersen, T. I. *et al.* Excitons in a reconstructed moiré potential in twisted WSe_2 /WSe_2 homobilayers. *Nat. Mater.* **20**, 480–487 (2021).

15. Rupp, A. *et al.* Imaging lattice reconstruction in homobilayers and heterobilayers of transition metal dichalcogenides. *ArXiv: 2306.14198* 24–29 (2023).
